# Enhanced Intracellular Stability and Translation Efficiency of mRNA Drugs by a 2‐arm mRNA Platform

**DOI:** 10.1002/advs.75244

**Published:** 2026-04-09

**Authors:** Xucong Teng, Jiahao Lin, Qiushuang Zhang, Xiangdong Zhang, Yicong Dai, Jinghong Li

**Affiliations:** ^1^ Center for BioAnalytical Chemistry Hefei National Laboratory of Physical Science at Microscale University of Science and Technology of China Hefei China; ^2^ Beijing Life Science Academy Beijing China; ^3^ New Cornerstone Science Laboratory Department of Chemistry Key Laboratory of Bioorganic Phosphorus Chemistry and Chemical Biology Tsinghua University Beijing China

**Keywords:** dendritic mRNA, 2‐arm mRNA, mRNA stability​, prolonged protein expression, ​mRNA therapy

## Abstract

The poly(A) tail deadenylation and transient protein expression of messenger RNA (mRNA) extremely hinder its therapeutic potential in genetic diseases, from which it follows that improving RNA stability and translation efficiency has emerged as a critical priority. In this study, we construct a 2‐arm mRNA via a streamlined modular assembly approach, characterized by a unique topology formed through the dimerization of two mRNA 3’ poly(A) tails. This distinctive architecture exhibits the capacity for efficiently recruiting poly(A)‐binding proteins (PABPs) to activate the eIF3‐eIF4F complex and promote highly efficient cap‐dependent translation, markedly improving 3’ tail stability and resistance to nuclease degradation, with an intracellular half‐life of up to 65 h. Furthermore, the 2‐arm mRNA sustains higher‐level protein expression for over two weeks in protein replacement therapy of hemophilic mice compared to linear mRNA. In conclusion, this work presents a novel 2‐arm mRNA platform that substantially enhances the translation capacity of mRNA, broadening its potential applications in mRNA‐based therapeutics, particularly for the treatment of genetic disorders.

## Graphical abstract

We constructed a 2‐arm mRNA, characterized by a unique topology formed through the dimerization of two mRNA 3’ tails. The 2‐arm mRNA improves 3’ tail stability and resistance to nuclease degradation, resulting in an intracellular half‐life of up to 65 h. This method substantially enhances the translation capacity of mRNA drugs.

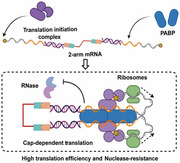

## 1 Introduction

mRNA therapy has achieved great success in the development and application of mRNA vaccines, owing to its advantages in easy synthesis, design, and modification [1, 2, 3, 4]. This has sparked researchers’ interest in developing it as a versatile therapeutic strategy for broader applications, including protein replacement, gene editing, and immunotherapy [5, 6, 7, 8, 9]. However, unlike mRNA vaccines, mRNA‐based protein replacement therapies require higher levels of protein expression to reach the therapeutic threshold [10, 11]. In vitro prepared linear mRNA has a short intracellular half‐life, which makes it difficult to achieve the expected translation efficiency and limits the broader application of mRNA‐based therapies [12, 13, 14].

Several strategies have been developed to enhance the stability and translation efficiency of mRNA [15]. Optimization and chemical modification of the sequences at the 5’‐untranslated region (5’‐UTR), coding sequence (CDS), and 3’‐untranslated region (3’‐UTR) have been proven to enhance the half‐life and translation efficiency of mRNA, but they still fall short of the requirements for mRNA therapy development [16, 17, 18, 19, 20]. Novel mRNA platforms, such as circular RNA (circRNA) and self‐amplifying RNA (saRNA), have also been explored to enhance overall protein yield and stability to some extent, though they come with notable limitations [21, 22]. The circRNA shows good resistance to exonucleases degradation due to the absence of free 3’‐end [23, 24]. However, its translation relies on internal ribosome entry sites (IRES), which generally exhibit lower translation efficiency compared with cap‐dependent translation mechanisms [25]. Moreover, base modifications are incompatible with circRNA, as they interfere with the RNA splicing process required for circularization and impede translation initiation [26, 27]. saRNA enables intracellular amplification of mRNA transcripts, thereby increasing overall target protein production and countering the effects of mRNA deadenylation [28, 29]. However, its replication process is uncontrollable and may generate double‐stranded RNA (dsRNA) by‐products. Moreover, conserved sequence elements essential for saRNA amplification are incompatible with m^1^ψ modification [30, 31, 32, 33]. This poses a risk of triggering innate immune responses in vivo. Therefore, further improvements in both the half‐life and translation efficiency of mRNA therapeutics continue to be of paramount importance.

Cap‐dependent translation initiation, as the most prevalent translation mechanism in eukaryotes, enables highly efficient protein expression, which relies on the assembly of the translation initiation complex (mainly composed of eIF4E and eIF4G) [34]. In particular, the recruitment and assembly of key translation initiation factors centered around the m^7^G cap at the 5' end of mRNA by eIF4E can significantly determine mRNA translation efficiency [34, 35]. Translation initiation complex can interact with poly(A)‐binding proteins (PABPs) in the cytoplasm, which bind to the 3’‐end poly(A) tail of mRNA. Therefore, they can form a pseudo‐closed‐loop structure [36]. Poly(A) binding protein cytoplasmic 1 (PABPC1) plays a dual role in this process: On the one hand, it initiates protein expression by interacting with the translation initiation factor eIF4G; on the other hand, it protects the 3' poly(A) tail from rapid deadenylation by shielding the poly(A) tail, thereby extending mRNA half‐life [34, 36, 37, 38]. Therefore, stabilizing the mRNA poly(A) structure and enhancing the interaction between poly(A) and PABPC1 are considered effective strategies for improving mRNA stability and translation efficiency. Recently, Wang and colleagues have synthesized mRNAs with unnatural topological structures of branched poly(A) tails using chemical synthesis and ligase‐based assembly strategies, demonstrating that such constructs significantly enhance mRNA stability and translation efficiency [39]. However, the complex synthesis approach and purification steps hinder large‐scale preparation and widespread application. Therefore, there is an urgent need to develop simple and efficient chemical modification strategies to improve mRNA stability and translation efficiency.

Herein, we developed a novel mRNA synthesis platform that coupled multiple mRNA tails to form dendritic products through click chemistry and nucleic acid hybridization. Based on rapid and simple synthetic steps, we synthesized various dendritic mRNAs with different anti‐nuclease chemical modifications and topological patterns. We further characterized their translation capacity and demonstrated that one variant, termed 2‐arm mRNA, exhibits the most sustained and efficient protein expression. With a half‐life of up to 65 h, 2‐arm mRNA prolongs protein expression in mice for over two weeks, and achieves superior therapeutic efficacy in a hemophilia mouse model compared with linear mRNA at the same dosage. The 2‐arm mRNA is expected to become a novel tool for mRNA‐based therapeutic strategies.

## 2 Results

### 2.1 Design and Construction of Dendritic mRNA

**FIGURE 1:**
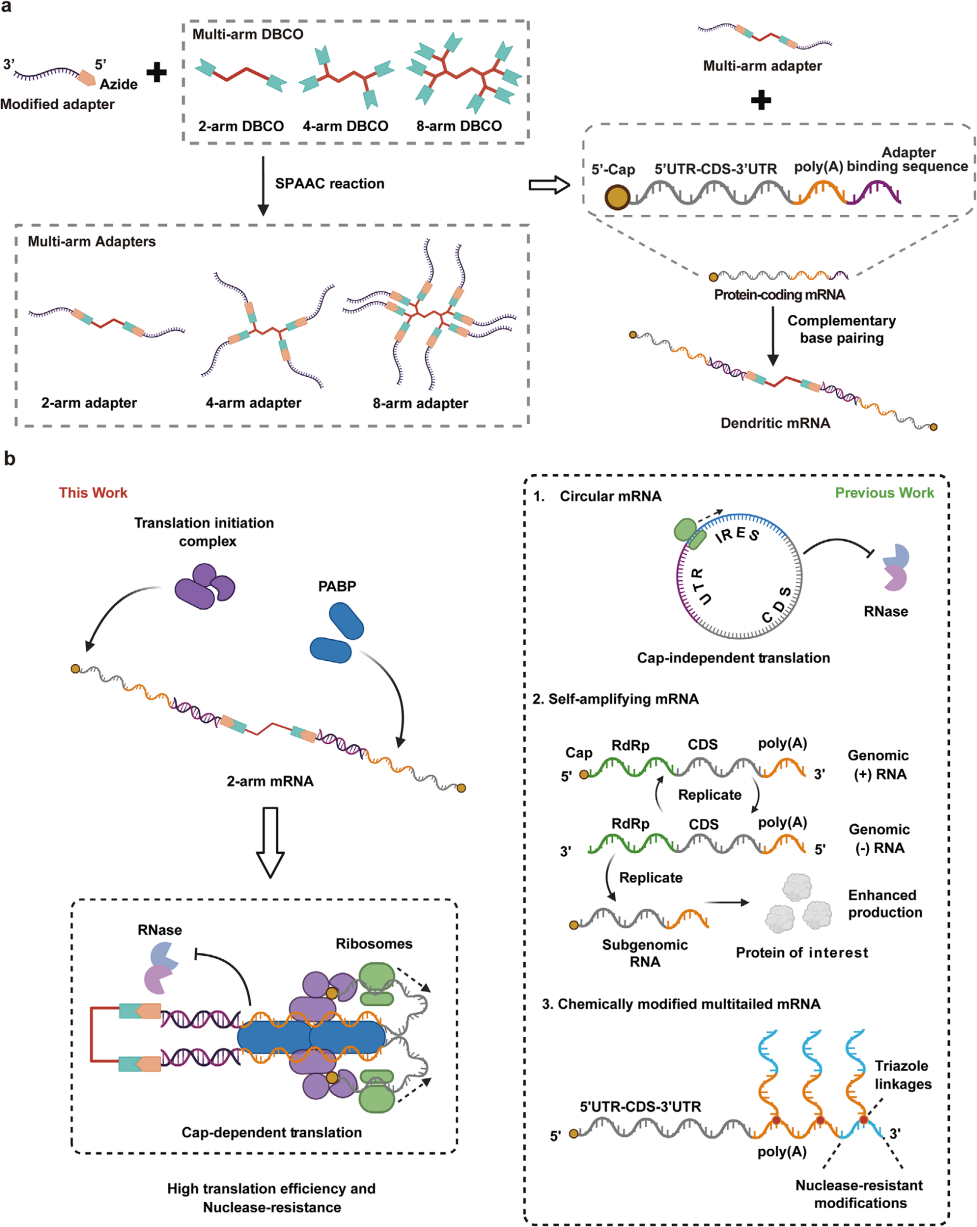
Design and construction of dendritic mRNA. (a) Schematic illustration of the synthesis of multi‐arm adapters and dendritic mRNA. Multi‐arm adapters served as the core scaffold for constructing dendritic mRNAs. The modified adapter was synthesized using unnatural bases and functionalized with an azide group at the 3’‐end. After HPLC purification, it was conjugated to multi‐arm DBCO via a SPAAC reaction to yield the final multi‐arm adapters. A protein‐coding mRNA sequence encoding the gene of interest, flanked by optimized 5’‐UTR and 3’‐UTR sequences, was ligated to the multi‐arm adapters through complementary base pairing to construct dendritic mRNA. (b) Overview of the principles of this RNA platform and previously reported systems.

We developed a modular assembly approach to construct dendritic mRNA. First, we synthesized a dendritic core scaffold. We prepared DNA adapters with an azide group at the 5’‐end, called modified adapters, and three types of PEG‐based multi‐arm dibenzocyclooctyne (DBCO) (including: 2‐arm DBCO, 4‐arm DBCO, and 8‐arm DBCO). These components were subsequently conjugated via the strain‐promoted azide‐alkyne cycloaddition (SPAAC) reaction to generate multi‐arm adapters [40, 41]. Then we designed a 26 nt sequence downstream of the 3’‐poly(A) of the mRNA that was completely complementary to the adapter to bind mRNA with the multi‐arm adapters via Watson–Crick base pairing. Finally, we obtained three types of constructs named 2‐arm mRNA, 4‐arm mRNA, and 8‐arm mRNA after conducting the methods mentioned above (Figure 1a). This strategy achieves simple and efficient assembly of mRNA and multi‐arm adapters into dendritic constructs while preserving the key functional elements in mRNA for cap‐dependent translation, including the 5’‐end m^7^G cap, optimized 5’/3’‐UTRs, CDS, and 3’‐poly (A) tail (Figure 1b) [42].

**FIGURE 2:**
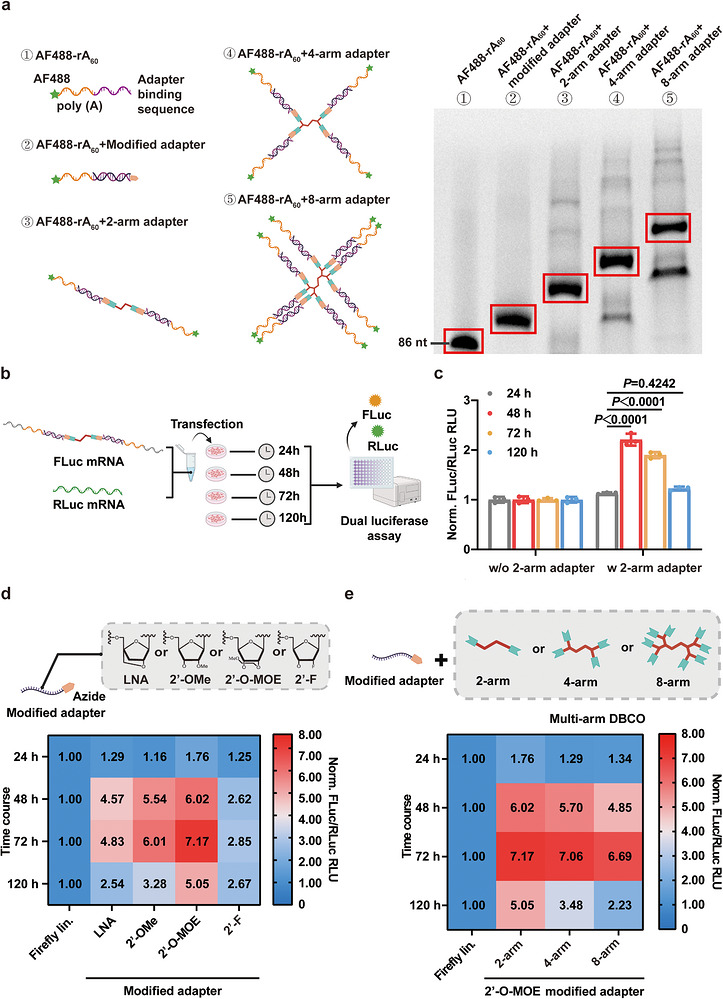
Functional verification and optimization of dendritic mRNAs. (a) Electrophoresis analysis of complementary pairing between AF488‐rA_60_ and multi‐arm adapters. AF488‐rA_60_, an RNA oligonucleotide containing a 60 nt poly(A) and adapter binding sequence, labeled with Alexa Fluor 488 at the 5’‐end, for simulating the complementary pairing process between the 3’‐end of mRNA and the multi‐arm adapter. Lane 1–5 in electrophoresis shows AF488‐rA_60_ and the products of AF488‐rA_60_ combining with different types of adapters, respectively. The expected band position of each product is highlighted with a red box. The proportion of the target dendritic constructs was quantified using Image J. (b) Schematic diagram of the dual‐luciferase assay for measuring the relative expression levels for linear mRNA and dendritic mRNA. Linear mRNA or dendritic mRNA encoding the same Firefly luciferase (FLuc) sequence was co‐transfected with linear Renilla mRNA. The intensities of FLuc and RLuc in the cell lysates were measured using a microplate reader over a period of 24 to 120 h, and the relative expression level of FLuc/RLuc was calculated. (c) Bar plots of the time‐course dual‐luciferase assay comparing the translation efficiency of mRNAs with and without the 2‐arm adapter scaffold. w/o, mRNA without 2‐arm adapter; w, mRNA with 2‐arm adapter. Data are presented as mean ± S.D. (n = 3 independent replicates). Statistical significance was assessed by one‐way ANOVA for comparisons across time points. (d) Evaluation of different unnatural nucleotides incorporated into modified adapters. Expression levels of dendritic mRNAs containing adapters with various unnatural nucleotides are displayed as a heatmap. (e) Evaluation of different multi‐arm adapter architectures (2‐arm, 4‐arm, and 8‐arm). Expression levels of dendritic mRNAs incorporating these multi‐arm adapters are shown in a heatmap. Firefly luciferase (FLuc) activity, normalized to RLuc and to a linear FLuc mRNA control, was measured at 24, 48, 72, and 120 h post‐transfection. The color scale of the heatmaps represents normalized FLuc/RLuc relative light units (RLU).

Subsequently, we validated the feasibility of this method. We used a single‐stranded RNA oligonucleotide with an Alexa Fluor 488 fluorescent label at the 5’ end, a 60 nt poly(A) and 26 nt adapter binding sequence at the 3’ end (AF488‐rA_60_) to simulate the complementary pairing process between the 3’‐end of mRNA and the multi‐arm adapter. We mixed AF488‐rA_60_ with three different multi‐arm adapters at the intended molar ratio (2:1 for 2‐arm adapter, 4:1 for 4‐arm adapter, and 8:1 for 8‐arm adapter) to initiate the SPAAC reaction. The target products were identified using 15% denaturing polyacrylamide gel electrophoresis (PAGE) and the proportion of the target dendritic constructs was quantified using Image J. The electrophoresis result indicated that the corresponding dendritic products were successfully synthesized, and the proportion of the target products reaches over 80% of the total products (Figure 2a). In the subsequent experiments, we employed the same experimental conditions to synthesize 2‐arm mRNA, 4‐arm mRNA, and 8‐arm mRNA. To further validate the successful synthesis of the dendritic mRNA constructs and quantify the overall synthetic yield and purity, we performed quantitative analysis of the target 2‐arm Firefly luciferase (FLuc) mRNA product in the crude reaction mixture using the Qsep1 Bio‐Fragment Analyzer coupled with gel gray value analysis (Figure S1). The overall synthetic yield of 2‐arm mRNA was calculated as 90.8 ± 0.03%, with final purity of more than 90%, and showed excellent batch‐to‐batch consistency in physicochemical properties and functional activity.

### 2.2 Functional Characterization and Optimization of Dendritic mRNA

After successfully synthesizing dendritic mRNA, we sought to investigate their translation capacity. We utilized the dual‐luciferase assay to measure the relative expression levels for linear mRNA and 2‐arm mRNA with the same FLuc sequences, compared with the linear Renilla (RLuc) mRNA co‐transfection control reporter over a period of 24 to 120 h (Figure 2b). We found that the 2‐arm FLuc mRNA maintained a higher relative luciferase expression level within 120 h compared with linear mRNA (Figure 2c). These results indicate that the dendritic mRNA contributes to prolonging the duration and increasing the yield of mRNA translation.

We next attempted to optimize the base structure of the modified adapter in dendritic mRNA. The DNA‐RNA hybrid double helix formed by complementary pairing between the 3’‐end of mRNA and the deoxyribonucleotides on the modified adapter is prone to digested by RNase H in living cells [43]. Therefore, we employed chemically modified unnatural nucleotides (Xenobiotic nucleic acid, XNA) to resist the RNase H and other ribonucleases degradation [43, 44], further enhancing the stability of dendritic mRNA. To this end, we synthesized 2‐arm adapters with unnatural nucleotides, including locked nucleic acids (LNA), 2’‐methoxy (2’‐OMe), 2’‐methoxyethoxy (2’‐O‐MOE), and 2’‐deoxy‐2’‐fluoro (2’‐F) nucleotides [43, 45]. Subsequently, we compared the expression level of 2‐arm mRNA with different XNA modifications using the dual‐luciferase assay. Within 48–120 h post‐transfection in HeLa cells, the 2‐arm mRNA with modified adapter composed of 2’‐O‐MOE nucleotides exhibited the highest relative expression level of FLuc, which was 5.05–7.17 fold higher than that of linear mRNA (Figure 2d; Figure S2a). These results indicate that the modified adapter composed of 2’‐O‐MOE bases enables 2‐arm mRNA to achieve better expression within 120 h post‐transfection.

We further optimized the number of arms in the dendritic structure. The relative expression levels of Fluc for 2‐arm mRNA, 4‐arm mRNA, and 8‐arm mRNA were measured using a dual‐luciferase assay over a time‐course of 24 to 120 h. The results demonstrated that, compared with mRNA with more dendritic topological structures, 2‐arm mRNA maintained the highest level of protein expression within 24–120 h post‐transfection, while increasing the number of arms that more than 2 arms does not significantly enhance the overall protein production of mRNA (Figure 2e; Figure S2b). Collectively, 2‐arm mRNA with 2’‐O‐MOE modified adapter exhibited the highest level of protein expression compared with linear mRNA, we used this form of 2‐arm mRNA in the subsequent experiments.

### 2.3 2‐arm mRNA has Enhanced Stability and Translation Efficiency

**FIGURE 3:**
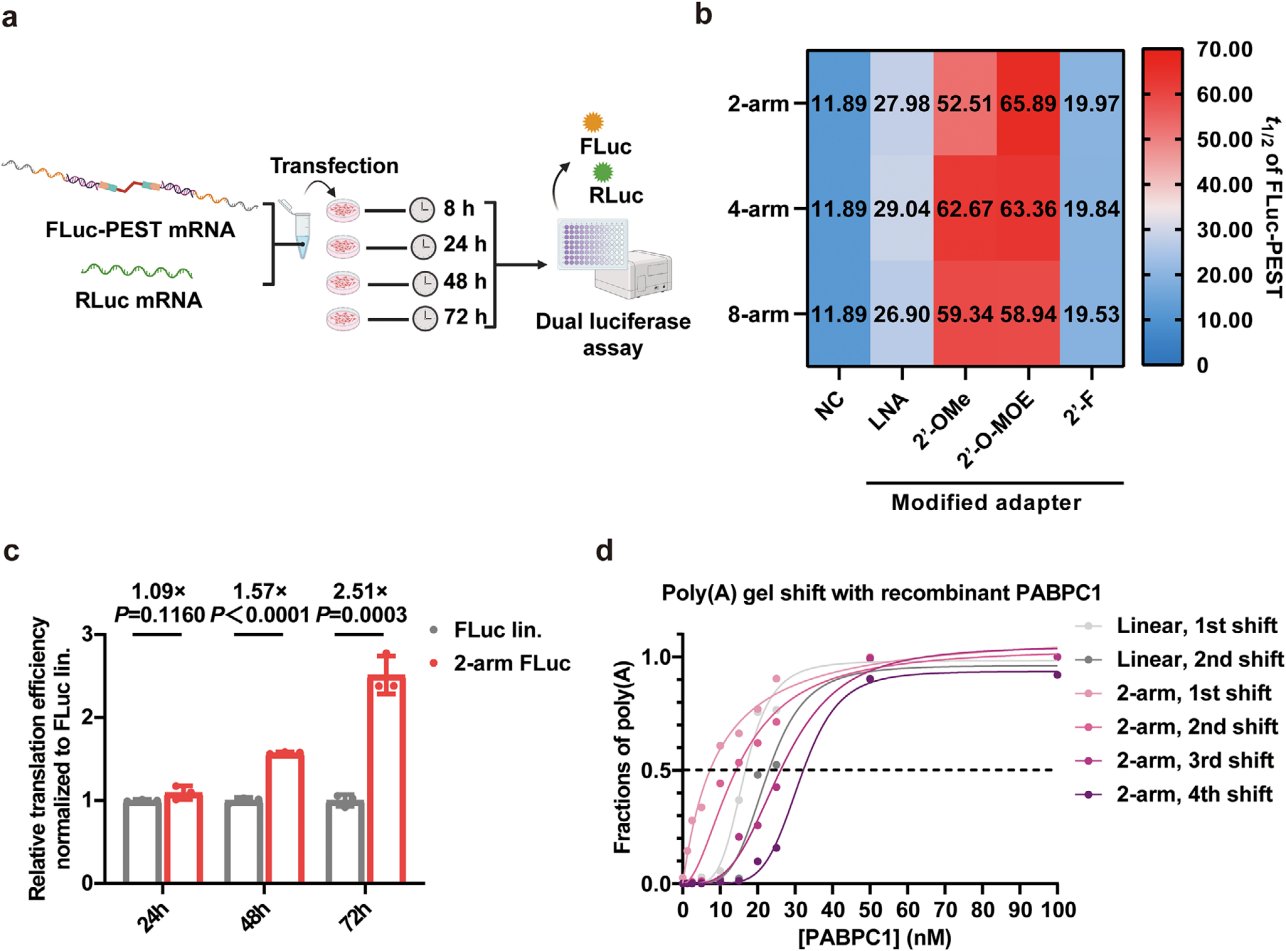
Mechanism of stability and translation efficiency for dendritic mRNAs. (a) Schematic illustration of kinetic profiling of FLuc‐degron (PEST) reporter in dendritic mRNAs. (b) Heatmap of FLuc‐PEST mRNA half‐life (*t*_1_/_2_) for different arm numbers and adapter modifications. Color scale represents *t*_1_/_2_ of FLuc‐PEST. (c) Time‐course comparison of the relative translation efficiencies (TE) of linear and 2‐arm FLuc mRNA.​ Relative translation efficiencies were measured by TE of 2‐arm mRNA normalized to TE of linear mRNA at 24, 48, and 72 h post‐transfection. Data are presented as mean ± S.D. (n = 3 independent replicates). Two‐tailed unpaired t‐test was carried out. (d) Quantification of gel‐shifting of linear and 2‐arm poly(A) at increasing concentrations of recombinant human PABPC1. The relationship between the percentage of poly(A) oligonucleotides bound to PABPC1 and the concentration of PABPC1 was plotted and fitted with Hill slope analysis for the dissociation constant (*K*_d_).

mRNA stability (half‐life, *t*_1/2_) and translation efficiency are key factors affecting the protein expression level of mRNA. To further elucidate the mechanism by which dendritic mRNA enhances translation capacity, we assessed their intracellular half‐life and translation efficiency. First, we characterized the translation kinetics of mRNA at different time points. Based on a previously reported strategy [39], we used a dual‐luciferase assay to measure the relative expression levels of Fluc from linear mRNA and dendritic mRNAs over a time‐course of 8–72 h with the Renilla luciferase (RLuc) mRNA as an internal transfection control, then calculating the half‐life of them (Figure 3a). The mRNA encoding FLuc luciferase was tagged with a degron‐tag (Firefly‐PEST), which significantly shortens the half‐life of the luciferase protein, allowing the luminescence signal to directly reflect the half‐life of the mRNA [46]. The luminescence decay kinetics showed that the 2’‐O‐MOE modified 2‐arm adapter promoted the greatest increase in mRNA *t*_1/2_. The *t*_1/2_ increased from 11.89 ± 0.4 h for linear mRNA to 65.89 ± 3.4 h for 2‐arm mRNA (Figure 3b, Figure S3a). The expression level of Firefly‐PEST encoded by 2‐arm mRNA was increased by 5.54‐fold compared with linear mRNA (Figure S3d). The luminescence decay kinetics of 4‐arm and 8‐arm adapter‐modified dendritic mRNAs also showed a similar trend, but were still lower than that of 2‐arm mRNA (Figure S3b,c). These results further confirmed that chemical modification and dendritic topology enhance the translation sustainability of 2‐arm mRNA by increasing its half‐life.

Additionally, we further employed RiboLace‐qPCR [47] to quantitatively analyze the translation efficiency of 2‐arm mRNA. This method can specifically capture ribosome‐bound mRNA to assess the translation levels of target genes [47]. We found that the RiboLace‐qPCR results were consistent with the trends observed in the cellular luciferase assay (Figure 3c). At 24 h post‐transfection, the difference in translation efficiency between 2‐arm mRNA and linear mRNA was not significant, suggesting that the introduction of chemical modifications and dendritic topology did not markedly affect mRNA translation efficiency during the early stages of transfection. However, at 48 h post‐transfection, the translation efficiency of 2‐arm mRNA was 1.57‐fold higher than that of linear mRNA. This trend became more pronounced, with the translation efficiency of 2‐arm mRNA being 2.51‐fold higher than that of linear mRNA at 72 h after transfection. These results suggest that 2‐arm mRNA remains structural stability over a longer period after transfection. This might be attributed to chemical modification and dendritic topology on 2‐arm mRNA, which preserves and enhances the function of the poly(A) structure, thereby better maintaining cap‐dependent translation initiation and improving the efficiency of translation.

### 2.4 Mechanisms Underlying the Enhanced Translation Capacity and Stability of Dendritic mRNA

We further verified whether 2‐arm mRNA promotes the initiation of mRNA translation by facilitating more efficient binding of its poly(A) tail with PABPC1, since the poly(A)‐PABPC1 interaction is a critical initiation step in cap‐dependent mRNA translation [37, 48]. We performed electrophoretic mobility shift assays (EMSA) and calculated the dissociation constant (*K*_d_) to quantify the interaction strength between PABPC1 protein and an RNA oligonucleotide containing a 30 nt poly(A) with an adapter binding sequence, labeled with Alexa Fluor 488 at the 5’‐end (linear AF488‐rA_30_) or corresponding 2‐arm AF488‐rA_30_. The dissociation constant (*K*_d_) for the first mobility shift of linear AF488‐rA_30_ and 2‐arm AF488‐rA_30_ was 16.6 and 9.0 nm, respectively. The *K*_d_ of linear AF488‐rA_30_ is consistent with the previously reported *K*_d_ value for the binding of linear poly(A) to PABP [49], while 2‐arm AF488‐rA_30_ exhibited a higher affinity for PABPC1. Subsequently, the poly(A) of 2‐arm mRNA exhibited a second mobility shift at 14.8 nm, followed by third and fourth shifts at 26.8 and 31.5 nm, respectively. In contrast, linear AF488‐rA_30_ only showed a second shift at 22.6 nm. These results indicate that dendritic poly(A) binds more PABPC1 proteins compared with linear poly(A) (Figure 3d, Figure S4). Given that the concentration of PABPC1 in the cytoplasm has been reported to be approximately 1 µm, which is at least three orders of magnitude higher than the *K*_d_ values [50]. We suppose that all poly(A) tails on dendritic mRNA may be saturated with PABPC1 binding in cells, leading to more efficient protein translation.

To evaluate the nuclease resistance of 2‐arm mRNA, we conducted in vitro enzymatic digestion assays to simulate the degradation of the mRNA 3’‐end by nucleases in vivo. We assessed the resistance of both linear and 2‐arm AF488‐rA_60_ constructs to degradation by the recombinant CAF1‐CCR4 complex. CAF1‐CCR4 is a major deadenylase complex in eukaryotic cells, which is able to completely degrade poly(A) tails that have been initially shortened to approximately 60 nt by PAN2‐PAN3 [51, 52, 53]. The degradation efficiency of the CAF1‐CCR4 complex against 2‐arm AF488‐rA_60_ was significantly reduced compared with that of linear AF488‐rA_60_ (Figure S5a,b), indicating that chemical modification and the dendritic topology effectively protect the poly(A) tail from CAF1‐CCR4‐mediated mRNA degradation.

To further evaluate the resistance of 2‐arm mRNA to other potential RNA degradation pathways in cells [54], we simulated the complex cytoplasmic environment using HeLa cell cytoplasmic lysates, and treated linear and 2‐arm AF488‐rA_60_ with the lysates, respectively, to assess their decay rates. Gel electrophoresis results showed that linear poly(A) exhibited significant degradation after 90 min of treatment with HeLa cytoplasmic lysates, whereas 2‐arm poly(A) maintained good stability even after 210 min of lysate treatment (Figure S5c,d). Collectively, the dendritic topology enhances the binding of the poly(A) tail of mRNA to PABPC proteins and protects the 3’‐tail of mRNA from nuclease attack.

### 2.5 2‐arm mRNA Relies on eIF4‐eIF3 Complex for Translation Initiation

**FIGURE 4:**
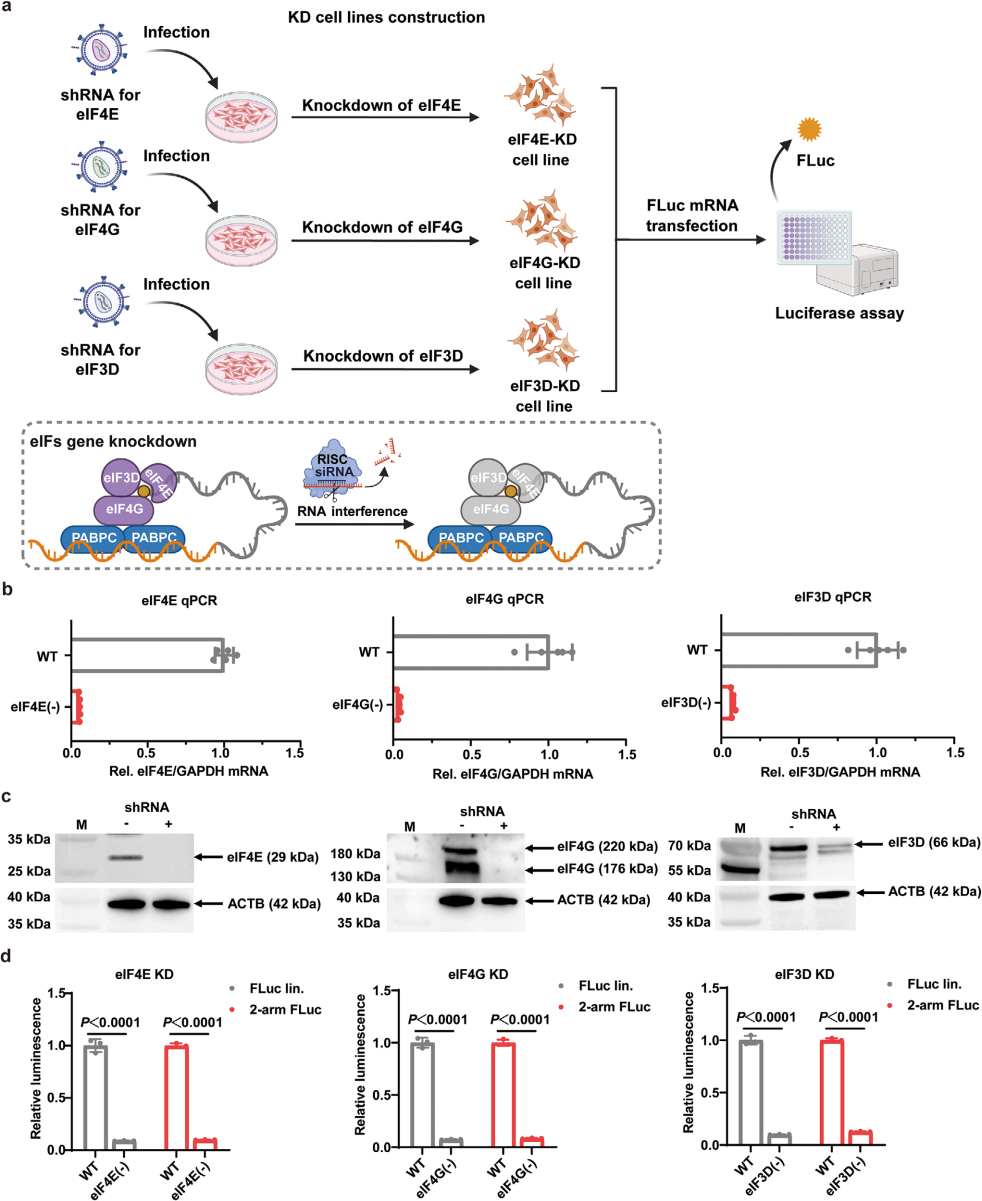
2‐arm mRNA relies on the canonical eIF4‐eIF3 translation initiation pathway.​​ (a) Schematic illustration of gene knockdown (KD) experiments for eIF4E/eIF4G/eIF3D genes and assessment of luciferase expressions in eIF4E/eIF4G/eIF3D KD Hela cells. (b) RT‐qPCR assay for quantifying the relative gene expression levels of eIF4E, eIF4G, and eIF3D normalized to GAPDH in wild‐type Hela cells (WT) and KD Hela cells. (c) Western blotting analysis of eIF4E, eIF4G, and eIF3D expression in WT and KD Hela cells. The uncropped blots are shown in Figure S6. (d) Luciferase expressions assay of WT and KD Hela cells. Linear FLuc mRNA or 2‐arm FLuc mRNA was transfected into these cells. FLuc activity was detected 6 h post‐transfection. Luciferase expressions were normalized to the luminescence level in WT cells for each construct. Data are presented as mean ± S.D. (n = 3 independent replicates). *P* values were calculated by two‐tailed unpaired t‐test.

Given that the canonical translation initiation of mRNAs relies on the interaction between the 3′‐end poly(A)‐PABPC1 complex and eukaryotic translation initiation factors (eIFs) [34, 35, 36, 37, 55], we further examined the dependency of 2‐arm mRNA translation on eIFs. We established Hela cell lines with eIF4E, eIF4G, and eIF3D gene knockdown (KD) by shRNA, respectively (Figure 4a). The eIF4E‐eIF4G complex serves as a core regulatory component in eukaryotic translation initiation, responsible for recognizing the 5’‐m^7^G cap of mRNA, interacting with PABPs on the 3’‐poly(A) tail of mRNA, and recruiting ribosomes [34, 35, 36, 37]. eIF3D, on the other hand, is a key downstream component of eIF4 that recruits the 40S ribosomal small subunit and promotes the formation of the 43S pre‐initiation complex [34, 55]. We validated the respective eIF‐knockdown cell lines via RT‐qPCR and Western blot assays (Figure 4b,c, Figure S6). Subsequently, we transfected linear mRNA and 2‐arm mRNA encoding Fluc into wild‐type and eIF‐knockdown HeLa cells. Luminescence assays revealed a marked decrease in the relative protein expression in 2‐arm mRNA‐transfected eIF‐knockdown HeLa cells compared with that of wild‐type controls (Figure 4d). These results indicated that 2‐arm mRNA initiates translation through the eIF4‐eIF3‐dependent mechanism.

### 2.6 2‐arm mRNA Enables Prolonged Protein Expression In Vivo

**FIGURE 5:**
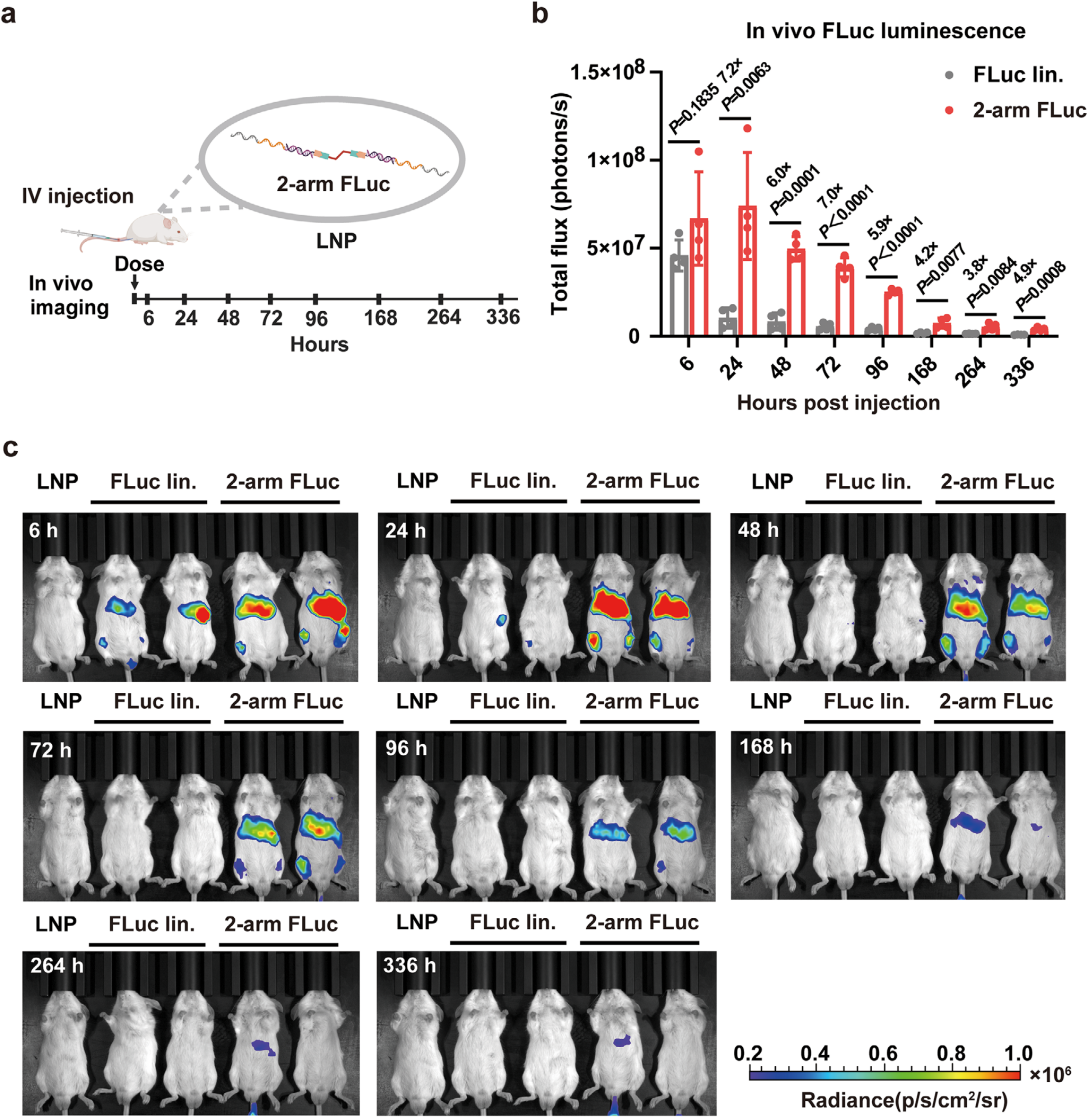
2‐arm mRNA extended duration of protein expression in vivo. (a) Schematic illustration of the 2‐week in vivo luminescence quantification assay. Equal amounts of LNP‐encapsulated linear/2‐arm FLuc mRNAs were administered in BALB/cJ mice using tail vein intravenous injection. (b) Quantification of the total flux of in vivo luminescence signals from FLuc mRNAs. Results were shown as mean ± S.D. (n = 4 mice per group). The *p* values were evaluated by one‐way ANOVA. (c) Representative in vivo bioluminescence images of mice from 6 to 336 h after injection of empty LNP/FLuc lin./2‐arm FLuc constructs. The color scale bar represents radiance (photons/s/cm^2^/sr).​.

Given the good performance of 2‐arm mRNA in cells, we further evaluated its performance in mice. Linear mRNA and 2‐arm mRNA were encapsulated in commercially available lipid nanoparticles (LNPs), respectively and the physicochemical characterization of them were performed. The encapsulation efficiency, hydrodynamic diameter, polydispersity index (PDI), and zeta potential of mRNA‐loaded LNPs all met the standard for in vivo delivery (Figure S7). Then the mRNA‐LNPs were administered via tail vein injection at a dose of 0.5 mg/kg (only the mRNA component was calculated). Protein expression levels of mRNAs in mice were then characterized and quantified using in vivo luminescence imaging (Figure 5a). We continuously monitored the expression levels of Fluc in mice at a time‐course from 6 to 336 h after administration. The luminescence signal of 2‐arm FLuc mRNA reached its peak within 6 to 48 h after administration, which was 6.0 to 7.2 times higher than that of linearized mRNA. Moreover, 2‐arm FLuc mRNA still maintained a higher luciferase luminescence signal than linear mRNA at later time points. Even two weeks after administration, the expression level of 2‐arm FLuc mRNA remained 4.9‐fold higher than that of linear mRNA (Figure 5b,c). These results indicate that 2‐arm mRNA can also achieve more sustained and efficient protein expression in vivo compared with linear mRNA.

### 2.7 2‐arm mRNA Enhances the Efficacy of Hemophilia Protein Replacement Therapy

**FIGURE 6:**
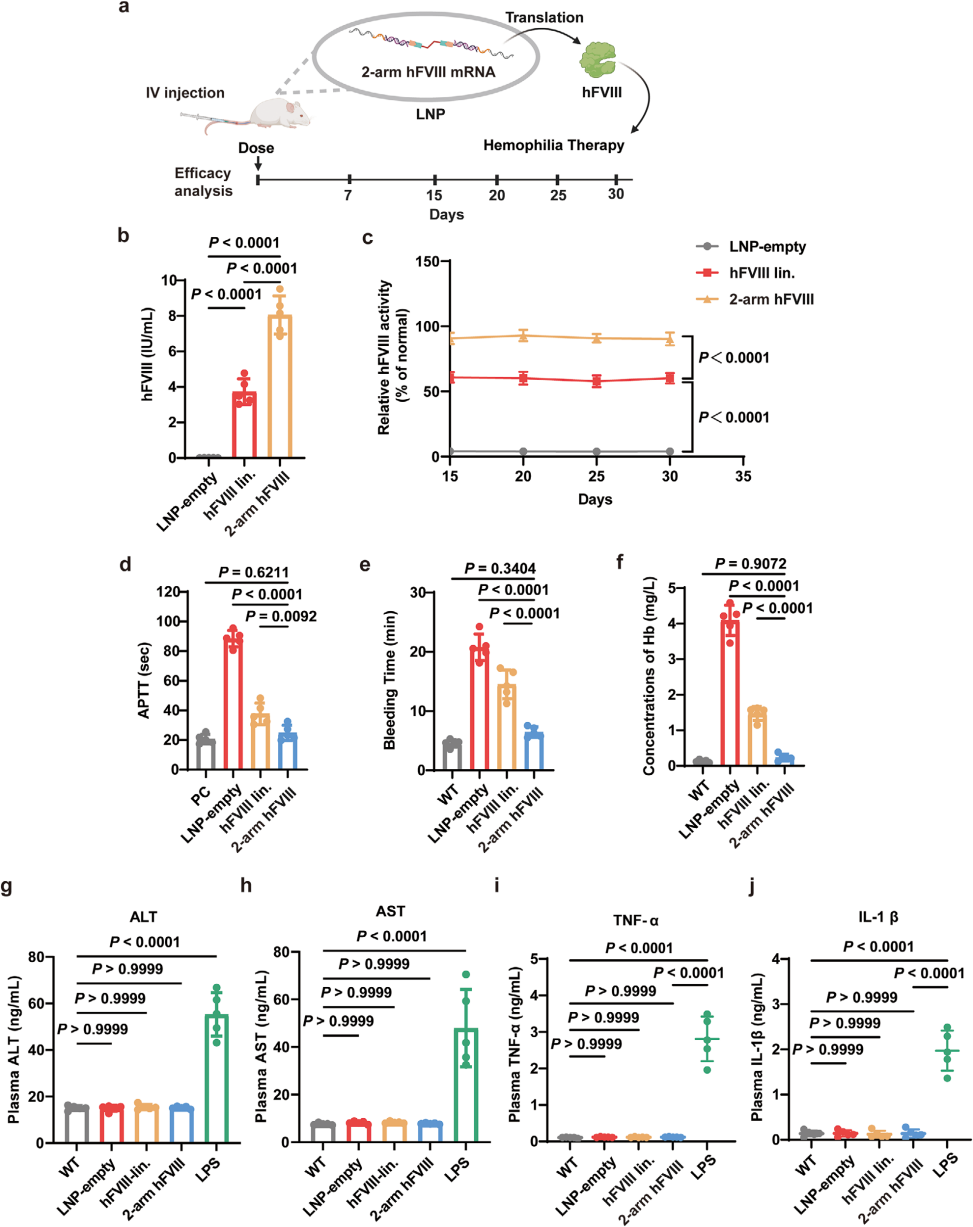
2‐arm FVIII mRNA enables prolonged hFVIII protein expression and the treatment of hemophilia in vivo. (a) Schematic diagram of the administration and characterization schemes for hemophilia treatment. Equal amounts of 2‐arm mRNA and linear mRNA (only the mRNA component was calculated) encoding human coagulation factor VIII (hFVIII) into FVIII gene knockout (FVIII KO) hemophilia mice. Blood was collected and analyzed at planned time points, and mouse organs were collected for immunohistochemical analysis a month post‐injection. (b) Quantification of hFVIII expression in mouse plasma by ELISA on the seventh day post‐injection. Results were presented as mean ± S.D. (n = 5 biological replicates for each group). The *p*‐values were calculated by one‐way ANOVA. (c) Kinetic profile of the relative protein activity of hFVIII over the time‐course of treatment. Mouse plasma was collected and analyzed from 15 to 30 days post‐injection. Results were presented as mean ± S.D. (n = 5 biological replicates for each group). The *p*‐values were calculated by one‐way ANOVA. (d–f) Analysis of parameters relative to the recovery level of coagulation function in mice. Activated partial thromboplasting time assay (APTT). Bleeding time. Concentrations of Hb at week four after protein‐replacement therapy. (g–j), Analysis of organ toxicity biomarkers and immunogenicity levels in plasmas. Levels of ALT and AST in plasma, levels of TNF‐α and IL‐1β in plasma on day seven post‐transfection were quantified by ELISA (n = 5 biological replicates; mean ± S.D.). *P* values were calculated by one‐way ANOVA.

Encouraged by the excellent translation performance of 2‐arm mRNA in mice, we attempted to verify its feasibility as a therapeutic platform for hemophilia. Hemophilia is a representative genetic bleeding disorder caused by defects in the genes related to the synthesis of coagulation factors [56, 57]. Patients suffer from severe coagulation disorders due to the deficiency of coagulation factors, among which the most common type is hemophilia A caused by the deficiency of coagulation factor VIII [56, 57]. Gene therapy to supplement coagulation factors and improve patient symptoms is one of the most effective treatment methods in clinical application at present [58, 59]. To verify the feasibility of 2‐arm mRNA for long‐term and sustainable treatment of hemophilia, we delivered equal amounts of 2‐arm mRNA and linear mRNA (only the mRNA component was calculated) encoding human coagulation factor VIII (hFVIII) into FVIII gene knockout (FVIII KO) hemophilia mice. Over the course of one month following administration, we continuously monitored indicators related to the hemophilia functional recovery to assess therapeutic efficacy (Figure 6a). ELISA results showed that mice treated with 2‐arm hFVIII mRNA reached the highest expression level of hFVIII, which was 2.2 times higher than that in mice treated with linear hFVIII mRNA at day 7 after administration (Figure 6b). From 15 to 30 days after 2‐arm hFVIII mRNA treatment, the relative activity of hFVIII in mouse plasma still remained at approximately 90% of the normal hFVIII activity (Figure 6c). In contrast, the relative hFVIII activity of linear mRNA decreased to about 60%. These results demonstrate that 2‐arm hFVIII mRNA enables a more sustained therapeutic effect for hemophilia in mice.

Additionally, we evaluated the recovery of coagulation function in hemophilia model mice. The hemophilia model mice were administered with hFVIII mRNA. Four weeks after administration, we utilized the activated partial thromboplastin time (APTT) assay to assess the recovery of intrinsic coagulation function [60]. We found that after treatment with 2‐arm hFVIII mRNA, the coagulation function of hemophilia mice was basically restored, and the APTT reached the normal range (Figure 6d). Subsequently, we performed a tail‐docking assay on the treated FVIII KO mice to evaluate the recovery of coagulation ability by measuring bleeding time. Compared with untreated and linear hFVIII mRNA‐treated FVIII KO mice, 2‐arm hFVIII mRNA treatment significantly improved bleeding symptoms, and the coagulation time showed no significant difference from that of wild‐type mice (Figure 6e). Since severe bleeding leads to a sharp increase in hemoglobin levels, we also measured plasma hemoglobin levels in the treated mice. The results indicated that hemoglobin concentrations in the plasma of mice treated with 2‐arm hFVIII mRNA returned to the normal range comparable to that of wild‐type mice (Figure 6f). These findings collectively demonstrate that 2‐arm hFVIII mRNA treatment can effectively restore coagulation function in the diseased mice.

Spontaneous bleeding in tissues is one of the important indicators for assessing the severity of hemophilia progression [58, 61]. Therefore, we further evaluated the spontaneous bleeding in various tissues of mice. We found a large number of red blood cells densely distributed in the interstitial tissues of the spleen, lungs, and kidneys in untreated hemophilia model mice, with an obvious Hemorrhagic focus (Figure S8). After human FVIII enzyme replacement therapy, the number of red blood cells in the interstitial tissues of hemophilic mice decreased, indicating a significant alleviation of spontaneous bleeding symptoms. Specifically, following treatment with 2‐arm hFVIII mRNA, the number of red blood cells in the interstitial tissues returned to a level comparable to that of wild‐type mice, which was much lower than that in the linear hFVIII mRNA treatment group, and no obvious hemorrhagic foci were observed (Figure S8). These results demonstrate that, at the same dosage, 2‐arm hFVIII mRNA therapy is more effective in reducing spontaneous bleeding symptoms caused by hemophilia.

Finally, the organ toxicity and immunogenicity of the drug were evaluated. Since intravenous delivery of LNP‐mRNA tends to target the liver and may be metabolized through the kidney, we measured the levels of alanine aminotransferase (ALT), aspartate aminotransferase (AST), Alkaline phosphatase (ALP) and blood urea nitrogen (BUN) in mouse plasma seven days after drug administration and found no significant differences in these indicators’ levels among wild‐type mice and mRNA‐treated groups (Figure 6g,h; Figure S9a,b) [62]. To rule out the possibility that the inherent inflammatory response of the FVIII KO may affect our accurate judgment of the biological safety of the drug, we evaluated the same biomarkers in healthy wild‐type mice (Figure S10). The results of healthy wild‐type mice were consistent with those of FVIII KO mice, demonstrating that our drug did not induce significant organ toxicity. Additionally, we assessed the immunogenicity of the drug by measuring the levels of tumor necrosis factor‐α (TNF‐α) and interleukin‐1β (IL‐1β) in plasma [63]. The levels of TNF‐α and IL‐1β in mice transfected with 2‐arm hFVIII mRNA showed no significant difference compared with wild‐type mice and other experimental groups, but were significantly lower than those in the positive control group injected with immunologic stimulant lipopolysaccharide (LPS) (Figure 6i,j) [64]. Furthermore, histopathological analysis of tissue sections from various organs of the treated mice revealed no obvious inflammatory reactions in organs (Figure S11). These results indicate that 2‐arm mRNA does not lead to long‐term organ toxicity or immunogenicity after in vivo delivery, demonstrating favorable biological safety and suitability for protein replacement therapy.

## 3 Discussion

Maintaining the long‐term stability of mRNA within cells and achieving sustainable and efficient protein expression are the main challenges currently faced by mRNA‐based therapeutic strategies [10, 11, 12, 13, 14]. Although various alternative RNA types, including circular RNA, self‐amplifying RNA, and multi‐tailed mRNA, have been developed, they still have some deficiencies in terms of immunogenicity, sequence designability, and synthetic difficulty [15, 21, 22, 39]. In this work, we developed a novel 2‐arm mRNA synthesis platform and achieved modular assembly of mRNA with other chemical elements via simple click reactions and complementary base pairing. Compared with the complex chemical synthesis or enzymatic ligation processes of other mRNAs previously developed, our synthesis strategy is more convenient and more suitable for large‐scale promotion and application (Figure 1). Furthermore, 2‐arm mRNA simultaneously maintains mRNA stability and enhanced translation efficiency without obvious immunogenicity. Importantly, these characteristics allow us to use lower doses of mRNA to achieve higher levels of functional protein expression in the gene therapy of hereditary diseases. In terms of clinical treatment, we have initially demonstrated the effectiveness of treating hemophilia using 2‐arm hFVIII mRNA, and achieving sustained protein expression for more than two weeks in mice without obvious immunogenicity during the treatment process. Therefore, 2‐arm mRNA is expected to provide a promising platform for the development of mRNA drugs.

Furthermore, we propose a potential translation initiation model for 2‐arm mRNA and a mechanism for enhanced translation capacity (Figure 1b). Unlike the classical linear mRNA translation initiation mechanism, during the translation initiation, the denser poly(A) tail of 2‐arm mRNA can enhance the recruitment efficiency for PABPs, facilitating their interaction with eIF4‐eIF3 complexes. Multiple PABP proteins may simultaneously interact with the two 3’‐poly(A) tails on the 2‐arm structure. This contributes to maintaining the stability of the mRNA poly(A) tails and improving the translation initiation efficiency of cap‐dependent translation, achieving efficient and sustained protein expression in vivo.

Overall, the 2‐arm mRNA transcends key limitations of existing mRNA technologies in regard to stability, translation efficiency, and immunogenicity, thereby offering an alternative mRNA‐based therapeutic platform. The 2‐arm mRNA is expected to warrant further investigation for its clinical application potential and may serve as a universal mRNA therapeutic modality to address unmet medical needs across genetic diseases, tumor immunotherapy, infectious disease prevention, and gene editing.

## 4 Methods

### 4.1 Plasmid Construction

The CDS of interest genes were respectively inserted into the optimized pcDNA3.1+ vector, which contained the T7 promoter sequence, a 5’ human α‐globin UTR sequence, a CDS sequence, a 3’ human α‐globin UTR sequence, a 100‐nt template‐encoded poly(A) tail sequence, an adapter binding sequence, and a BamHI restriction site. The plasmids were transformed into TOP10 chemically competent cells (Yeasen, 11801ES80). Single colonies were screened and expanded for the amplification of plasmids. The plasmids were extracted and purified using the EndoFree Maxi Plasmid Kit V2 (Tiangen, DP120), and the accuracy of the plasmid sequence was verified by agarose gel electrophoresis and Sanger sequencing. The FLuc and RLuc constructs were derived from the pmirGLO dual‐luciferase miRNA Target Expression Vector (Promega, E1330). The RLuc construct was constructed without cloning into the optimized vector. The FLuc‐PEST sequence was adapted from a plasmid generated by Xiao Wang's laboratory (Addgene, Plasmid#200396). The sequence information of hFVIII mRNA (NM_000132.4) was obtained from NCBI. All constructs mentioned above were synthesized by General Biology, Anhui, China. Sequence information was listed in Table S1.

### 4.2 mRNA Synthesis, Purification, and Characterization

Plasmids for mRNA preparation were linearized using BamHI‐HF (NEB, R0136S). The linearized plasmids were characterized by agarose gel electrophoresis, and the target product was recovered and purified using the FastPure Gel DNA Extraction Mini Kit (Vazyme, D301‐01). The target mRNAs were synthesized in vitro using the T7 High Yield RNA Synthesis Kit for Co‐transcription (low dsRNA) (Yeasen, 10634ES60), and the cap analog Cap1 GAG was added through co‐transcription capping. All UTPs in the mRNA were replaced with N^1^‐methyl pseudouridine‐5'‐triphosphate. After in vitro transcription, the products were digested with DNase I to remove the residual DNA template and purified using the Monarch Centrifugal Filter Columns RNA Purification Kit (NEB, T2050L). The concentration of mRNA was measured using the Qubit RNA XR Assay Kit (ThermoFisher Scientific, Q33224). The products were subjected to nucleic acid electrophoresis in agarose gel electrophoresis to confirm that the size of the products was correct. The electrophoresis results were observed using a gel imaging system (BLT, GelView 6000Plus).

### 4.3 Synthesis and Characterization of Dendritic mRNA/poly(A) Oligonucleotides

All oligonucleotides and the chemical modifications on the bases were synthesized by General Biology, Anhui, China. Information of oligos and the chemical modifications are listed in Table S2. Modified adapters with azide modification at 5'‐end were mixed with three types of multi‐arm DBCO (purchased from Xinyanbomei Biological Technology, Shannxi, China; DBCO‐PEG‐DBCO, MW: 1000, X‐GF‐0295‐1k; 4‐arm PEG DBCO, MW: 2000, X‐GF‐0113‐2K; 8‐arm PEG DBCO, MW: 10000, X‐GF‐0115‐10K) at specific molar ratios (2:1 for DBCO‐PEG‐DBCO, 4:1 for 4‐arm PEG DBCO, and 8:1 for 8‐arm PEG DBCO), and incubated at 37°C for 1 h in 1× SPAAC reaction buffer (20 mm HEPES pH 7.5, 100 mm NaCl, 0.5 U/µ L RNase Inhibitor). In this way, three types of multi‐arm adapters (2‐arm adapter/4‐arm adapter/8‐arm adapter) were synthesized. They were then mixed with mRNA or poly(A) oligonucleotides at different molar ratios (1:2 for 2‐arm adapter, 1:4 for 4‐arm adapter, and 1:8 for 8‐arm adapter) and reacted at 37°C for 2 h in 1× SPAAC reaction buffer. The products were subjected to nucleic acid electrophoresis in 15% Nucleic Acid Polyacrylamide Preformed Gel (Solarbio, NG01510‐S), and the electrophoresis results were observed using the gel imaging system (BLT, GelView 6000Plus). The overall synthetic yield and purity of 2‐arm FLuc mRNA was analyzed using Qsep1 Bio‐Fragment Analyzer (Bioptic) and Image J.

### 4.4 Cell Culture, mRNA Transfection and Luciferase Assay

Human HeLa cells were provided by the Cell Resource Center, Institute of Basic Medical Sciences, Chinese Academy of Medical Sciences/Peking Union Medical College (CAMS/PUMC). The cells were cultured in DMEM (ThermoFisher Scientific, 11995065) supplemented with 10% FBS (Yeasen, 40131ES76), 1× GlutaMAX Supplement (ThermoFisher Scientific, 35050061), 1× MEM Non‐Essential Amino Acids Solution (ThermoFisher Scientific, 11140050), and 1× penicillin‐streptomycin (Yeasen, 60162ES76). Cells were cultured and maintained at 37°C, 5% CO_2_, and passaged at a ratio of 1:5 every 2–3 days. mRNAs were transfected into cells at equal amounts using Lipofectamine MessengerMAX transfection reagent (ThermoFisher Scientific, LMRNA015), and cells transfected with the corresponding mRNAs were collected at the planned time points. The collected cells were treated with the Dual One Step Luciferase Reporter Gene Assay Kit (Yeasen, 11405ES80), and the expression ratio of FLuc/RLuc was calculated according to the protocal provided by manufacturer.

### 4.5 FLuc‐PEST mRNA Luminescence Decay Kinetics Assay

Based on a previously reported strategy [39], we used a dual‐luciferase assay to measure the half‐life (*t*_1/2_) of mRNA. Linear FLuc‐PEST mRNA and various dendritic FLuc‐PEST mRNAs were co‐transfected into Hela cells with RLuc mRNA using Lipofectamine MessengerMAX transfection reagent (ThermoFisher Scientific, LMRNA015) at equal amounts. The relative expression level of FLuc/RLuc in each group was detected using the Dual One Step Luciferase Reporter Gene Assay Kit (Yeasen, 11405ES80) at 8, 24, 48, and 72 h post‐transfection, respectively (RLuc acts as an internal transfection control for normalization). The half‐life (*t*_1/2_) of Firefly‐PEST was calculated by fitting the normalized FLuc activity decay to a first‐order kinetic model [lny = ‐kx + b (least squares)], where k is the degradation rate obtained from the slope, and *t*_1/2_ was calculated as ln (2)/k.

### 4.6 RiboLace‐qPCR for Measuring mRNA Translation Efficiency

Human Hela cells were transfected with linear FLuc mRNA or 2‐arm FLuc mRNA. At 24, 48, and 72 h post‐transfection, cells from different groups were trypsinized, and 1 × 10^6^ cells were resuspended in 1×PBS buffer (pH 7.2–7.4) (Solarbio, P1020) containing 500 µm 3P [47]. The suspension was incubated at 37°C for 5 min in a metal bath heater. The treated cells were then washed twice with RIP lysis buffer without Triton X‐100 (20 mm Tris‐HCl, pH 7.5, 150 mm NaCl, 5 mm MgCl_2_, 1 mm DTT). Subsequently, the cells were resuspended in 50 µL RIP lysis buffer containing 1% Triton X‐100 in a 1.5 mL centrifuge tube and incubated on ice for 30 min, with gentle tapping of the tube every 5 min to ensure complete lysis. Next, 20 µL streptavidin magnetic beads (ThermoFisher Scientific, 11206D) were thoroughly mixed with the lysate. The beads were washed with 50 µL bead‐washing buffer (5 mm Tris‐HCl, pH 7.5, 1 m NaCl, 0.5 mm EDTA, 0.05% Triton X‐100). The tube was placed on a magnetic separation rack and left for 1 min until the solution became clear, then discarded the supernatant to complete one wash. This washing step was repeated three times. The beads were then washed once with 50 µL bead‐solution buffer A (50 mm NaCl, 100 mm NaOH, 0.1% Triton X‐100) and once with 50 µL bead‐solution buffer B (100 mm NaCl, 0.1% Triton X‐100). The beads were resuspended in 50 µL blocking buffer (20 mm Tris‐HCl, pH 7.5, 150 mm NaCl, 5 mm MgCl_2_, 1 mm DTT, 0.1% Triton X‐100, 1% BSA) and placed on a rotator at 4°C, 1 h for blocking. The beads were then resuspended in 150 µL of blocking buffer, mixed well, and added to the centrifuge tubes containing the cell lysates. The tubes were incubated on a thermal mixer at 4°C, 600 rpm for 2 h. The tubes were then removed from the thermal mixer, briefly centrifuged, and placed on the magnetic separation rack for 1 min until the solution cleared. The beads were washed three times with 200 µL washing buffer (10 mm Tris‐HCl, pH 7.5, 150 mm NaCl, 1% Triton X‐100, RNase Inhibitor). Subsequently, the beads were resuspended in 200 µL PK buffer (100 mm Tris‐HCl, pH 7.5, 50 mm NaCl, 10 mm EDTA, 1% SDS) containing 2 µL proteinase K (ThermoFisher Scientific, 25530049). After thorough mixing, the tube was incubated at 55°C and 1100 rpm on a constant‐temperature shaker for 1 h. RNA was then extracted using ethanol precipitation and resuspended in 20 µL DEPC‐H_2_O, yielding the actively translating mRNA (RNC mRNA, Ribosome Nascent‐chain Complex mRNA) from the cells. In parallel, total RNA was extracted directly from additional replicate samples. Subsequently, RT‐qPCR was conducted on both the extracted RNC mRNA and total RNA, using the housekeeping gene Actin‐β as an internal reference to calculate the relative expression of the FLuc. The data were analyzed using Excel (Microsoft Corp.) and GraphPad Prism 9 (GraphPad Software). The translation efficiency (TE) was calculated as follows:

(1) $$\mathrm{Relative}\;\mathrm{TE}\;\%=\frac{\mathrm{Relative}\;\mathrm{RNC}\;\mathrm{mRNA}\;\mathrm{expression}}{\mathrm{Relative}\;\mathrm{total}\;\mathrm{mRNA}\;\mathrm{expression}}\;\times 100$$

Normalization of the TE of FLuc mRNA in each group was performed using linear FLuc mRNA as the control at different time points.

### 4.7 Gel Mobility Shift Assay (EMSA)

The ORF mammalian expression plasmid (Sino Biological Inc., HG13781‐ CH), encoding full‐length Human PABPC1 (NP_002559.2; C‐terminal His‐tagged), was transformed into BL21 (DE3) chemically competent cells (Yeasen, 11804ES80) and cultured in Luria‐Bertani medium containing 50 µg/mL kanamycin at 37°C and 200 rpm. When the OD600 of the E. coli reached 0.6–0.8, IPTG was immediately added, and the cells were incubated at 18°C and 200 rpm overnight to induce the expression of PABPC1‐His fusion protein. The cells were harvested at 4°C and 7500× g, and lysed with lysis buffer [10 mm Na_2_HPO_4_ (pH 7.4), 1.8 mm KH_2_PO_4_, 637 mm NaCl, 2.7 mm KCl, 10% glycerol, 1 mm phenylmethylsulfonyl fluoride, 0.5 mm 4‐(2‐aminoethyl) benzenesulfonyl fluoride hydrochloride, 0.15 µm aprotinin, 1 µm E‐64, and 1 µm leupeptin] and 0.15% polyethyleneimine. The cells were then sonicated, and the debris was removed by centrifugation at 15000× g. The supernatant was purified and eluted using His‐tagged protein purification magnetic beads (Yeasen, 20561ES25) according to the manufacturer's instructions. The purified protein was concentrated using an Amicon Ultra filter with a 10 kDa MWCO (Millipore, UFC5010) and quantified using the Qubit Protein Broad Range Assay kit (ThermoFisher Scientific, Q33211). The concentrated PABPC1‐His protein was serially diluted to different concentrations and incubated with 5’Alexa Fluor 488‐labeled 2‐arm/linearized poly(A) oligonucleotides in reaction buffer (10 mm Na_2_HPO_4_, 1.8 mm KH_2_PO_4_, 137 mm NaCl, 2.7 mm KCl, 5 mm MgSO_4_, and 1 mm DTT) at 4°C for 2 h. Electrophoretic mobility shift assays were performed using 10% EMSA PAGE gels. The Alexa Fluor 488‐labeled oligonucleotides were imaged using a gel imaging system (BLT, GelView 6000Plus), and the gray values of each band were quantified using Image J. Subsequently, the *K*_d_ value of the binding of poly(A) oligonucleotides to PABPC1 was calculated. The relationship between the percentage of poly(A) oligonucleotides bound to PABPC1 and the concentration of PABPC1 was plotted using GraphPad Prism with Hill slope analysis for the specific binding of PABPC1 to poly(A).

### 4.8 In Vitro Poly(A) Degradation Assay

The codon‐optimized DNA sequences encoding full‐length CCR4 (NP_001273719)/CAF1 (NP_001309021) were cloned into pET‐15b vector from GenScript. Plasmids encoding CAF1 and CCR4 were co‐transformed into BL21 (DE3) chemically competent cells (Yeasen, 11804ES80) and co‐expressed as N‐terminal 6×His‐tagged fusion proteins in Luria Bertani medium at 37°C. When the OD600 of the E. coli culture reached 0.6–0.8, isopropyl β‐D‐1‐thiogalactopyranoside (IPTG) was added to a final concentration of 1 mm, followed by overnight incubation at 18°C, 200 rpm. Cells were lysed in Lysis buffer containing 50 mm HEPES pH 7.5, 300 mm NaCl, and 25 mm imidazole, supplemented with EDTA‐free protease inhibitor cocktail, 5 µg/mL DNase I, 1 mg/mL lysozyme, and 2 mm DTT. The lysate was purified and eluted using His‐tag protein purification magnetic beads (Yeasen, 20561ES25). Protein was eluted in a buffer containing 10 mm HEPES pH 7.5, 200 mm NaCl, supplemented with 0.5 mm PMSF and 2 mm DTT, and then concentrated using an Amicon Ultra filter (10 kDa MWCO, Millipore, UFC5010).

For in vitro poly(A) degradation assays, 5’‐Alexa Fluor 488 labeled 2‐arm/linear poly(A) oligonucleotides were incubated at 37°C with either HeLa cell cytoplasmic extract or 100 nM CCR4/CAF1 in a reaction buffer containing 20 mm Tris‐HCl pH 7.5, 150 mm NaCl, 2 mm MgCl_2_, 1 mm DTT. Every 30 min, 5 µL of the reaction mixture was taken and mixed with an equal volume of 2× RNA loading buffer (denatured) (Solarbio, R1055) to terminate the reaction, followed by denaturation at 70°C for 5 min and immediate ice bath for 5 min. RNA samples were resolved on 15% nucleic acid polyacrylamide preformed gel(Solarbio, NG01010‐S), and results were analyzed using a gel imaging system (BLT, GelView 6000Plus).

### 4.9 Construction of eIF‐knockdown cell lines using lentivirus

The shRNA lentivirus plasmids targeting human eIF4E, eIF4G, and eIF3D genes were obtained from the Tsinghua University Gene Library Platform. The information of shRNA sequences is shown in Table S3. These constructs were co‐transfected with the packaging plasmid (psPAX2) and the envelope plasmid (pMD2.G) into 293T cells to package lentiviruses. Viral supernatants were collected 48–72 h after transfection, concentrated by ultracentrifugation, and the viral titer (TU/mL ≥1 × 10^8^) was determined. Hela cells were cultured to the logarithmic growth phase and infected with the shRNA‐packaged lentiviruses at the optimal multiplicity of infection (MOI), with the addition of 8 µg/mL polybrene (Yeasen, 40804ES76) to enhance infection efficiency. After 12 h of infection, the medium was replaced with complete medium, and the cells were cultured for an additional 48 h. Subsequently, 1 µg/mL puromycin (Yeasen, 727136ES01) was added for continuous selection. The puromycin‐containing medium was refreshed every 3–5 days until all cells in the negative control group (uninfected with lentivirus) died, while the experimental groups formed positive polyclonal cell populations. Finally, the knockdown efficiency of the eIF genes in each stable cell line was validated at both the transcriptional and translational levels using RT‐qPCR and Western blot, respectively. RT‐qPCR primers were purchased from Beyotime (Human EIF4E qPCR Primer Pair, Beyotime, QH02545S; Human EIF4G1 qPCR Primer Pair, Beyotime, QH02557S; Human EIF3D qPCR Primer Pair, Beyotime, QH06677S).

### 4.10 Western Blotting

Total proteins were extracted from HeLa cells using RIPA lysis buffer (Yeasen, 20115ES60) containing 1 mm PMSF (Beyotime, ST507‐10 mL) and quantified with the Qubit Protein BR Assay kit (ThermoFisher Scientific, Q33211). Equal amounts of total protein from the control group and different treatment groups were separated by SDS‐PAGE using a 4%–20% Precast Protein Plus Gel (Yeasen, 36264ES10) and transferred onto a PVDF membrane. GoldBand Plus 3‐color Regular Range Protein Marker (10–180 kDa) (Yeasen, 26616ES72) was used as a marker. The membrane was incubated with the corresponding primary antibodies at 4°C for 1 h [eIF4E (C46H6) Rabbit mAb (CST, 2067T), eIF4G (C45A4) Rabbit mAb (CST, 2469T), and eIF3D (E4I4S) Rabbit mAb (CST, 40765T), β‐actin, Rabbit mAb (Yeasen, 30102ES60)] after blocking. Following washing with 1× TBST, the membrane was incubated with an Anti‐rabbit IgG, HRP‐linked secondary antibody (CST, 7074S) at 4°C for 1 h. Finally, the PVDF membrane was developed using a chemiluminescent ultrasensitive detection kit (Yeasen, 36208ES76) and analyzed with a gel imaging system (BLT, GelView 6000Plus).

### 4.11 Physicochemical Characterization of mRNA‐LNPs

10 µg of 2‐arm and linearized hFVIII mRNA (only the mRNA component was calculated) were encapsulated in LNPs. The hydrodynamic diameter, PDI, and zeta potential of mRNA‐LNPs were measured by dynamic light scattering (DLS) using a Zetasizer Ultra instrument (Malvern Panalytical). For size and PDI detection, LNPs were diluted with nuclease‐free ultrapure water to an appropriate concentration, and loaded into a disposable polystyrene cuvette. Measurements were performed at a scattering angle of 90° and a constant temperature of 25°C. For zeta potential detection, diluted LNP samples were loaded into a folded capillary zeta cell, and measurements were performed under the same temperature and equilibrium conditions. The average values of three independent preparation batches were used for statistical analysis.

The encapsulation efficiency (EE) of linear and 2‐arm hFVIII mRNA in LNPs was quantified using the Quant‐iT RiboGreen RNA Assay Kit (Thermo Fisher Scientific, R11490) following the manufacturer's instructions. Briefly, two equal aliquots of each LNP sample were prepared: one aliquot was treated with 1% (v/v) Triton X‐100 to disrupt the LNP lipid bilayer and release the total encapsulated mRNA, while the other aliquot was diluted with nuclease‐free water (without Triton X‐100) to measure the concentration of unencapsulated free mRNA in the external aqueous phase. A standard curve of linear hFVIII mRNA with gradient known concentrations was prepared in parallel for each assay. The fluorescence intensity of each sample was measured at an excitation wavelength of 480 nm and an emission wavelength of 520 nm using a multi‐mode microplate reader (Thermo Fisher Scientific, Varioskan LUX). The encapsulation efficiency was calculated using the following formula:

(2) $$\begin{array}{ccc} & & \mathrm{Encapsulation}\;\mathrm{Efficiency}\;\%\\ & & \;\;=\frac{\mathrm{Total}\;\mathrm{mRNA}\;\mathrm{concentration}\;-\;\mathrm{Free}\;\mathrm{mRNA}\;\mathrm{concentration}}{\mathrm{Total}\;\mathrm{mRNA}\;\mathrm{concentration}}\\ & & \;\times \;100 \end{array}$$

Each sample was measured in triplicate technical replicates, and the average values of three independent preparation batches were reported.

### 4.12 Experimental Animals

In this study, BALB/cJ mice (male, approximately 6–8 weeks old) used for in vivo FLuc assays, as well as male C57BL/6JGpt and C57BL/6JGpt‐F8^em430Cd5d452^ mice (male, approximately 6–8 weeks old) used for hemophilia protein replacement therapy experiments and drug toxicity analyses, were all purchased from GemPharmatech Co., Ltd. The animals were housed in a specific pathogen‐free (SPF) barrier environment, with five mice per cage, under controlled conditions of 20 ± 3°C, 40%–60% relative humidity, and a 12‐h light‐dark cycle. Food and water were provided ad libitum. All animal experimental protocols were approved by Tsinghua University's Institutional Animal Care and Use Committee (IACUC) with the Animal Protocol number 23‐LJH4.

### 4.13 In Vivo Delivery of Firefly Luciferase mRNA and Animal Imaging

Equal amounts of 2‐arm and linearized FLuc mRNA (only the mRNA component was calculated) encapsulated by LNPs were administered to mice via tail vein injection. The in vivo expression level of FLuc was quantitatively measured at predetermined time points using a small animal in vivo 3D imaging system (BLT, AniView Kirin). D‐luciferin potassium salt (Yeasen, 40902ES03) was diluted in PBS and injected intraperitoneally into the mice at a substrate concentration of 150 mg/kg per mouse. The luminescence intensity of luciferase was quantitatively analyzed using the BLT AniView Kirin imaging software. Mice injected with empty LNPs served as the negative control, and their luciferase luminescence intensity was subtracted as background in each measurement.

### 4.14 Evaluation of the Therapeutic Efficacy of hFVIII mRNA in Hemophilia Treatment

Lipid nanoparticles (LNPs) were prepared by mixing equal amounts of 2‐arm and linear FLuc mRNA (only the mRNA component was calculated) with in vivo‐jet RNA+ mRNA transfection reagent (Polyplus, 101000122) in mRNA buffer. These LNPs were administered to F8 KO mice via tail vein injection. Mice injected with empty LNPs served as the negative control, while wild‐type C57BL/6JGpt mice or standard plasmas from healthy humans were used as positive controls. Blood was collected from the mice at specified time points, and plasma was extracted to monitor various indicators related to hemophilia functional recovery. The human coagulation factor VIII (hFVIII) content in mouse plasma was quantified using the Human Coagulation Factor VIII (F8) ELISA Kit (Biosen, BES1995K). Relative activity changes of hFVIII were measured using the Activated Coagulation Factor VIII (FACTOR VIIIa) Fluorescent Quantitative Detection Kit (GIVEI, G&VS50486.2). Activated partial thromboplastin time (APTT) was determined using the APTT Colorimetric Assay Kit (GIVEI, G&VS10178.2), and post‐treatment hemoglobin levels in mice were measured with the Hemoglobin Detection Kit (Biosen, BES2035CMT). All assays were performed according to the manufacturers’ instructions.

### 4.15 Tail‐docking Experiment

Four weeks after hFVIII mRNA treatment, the experimental mice were anesthetized, and their tails were disinfected before the distal portion (approximately 2 mm in diameter) was cut. The tail was then immersed in physiological saline to collect blood for 30 min, and the bleeding time was recorded. C57BL/6JGpt‐F8 mice receiving empty LNP delivery served as the negative control, while normal wild‐type (WT) mice were used as the positive control. The bleeding times of the experimental groups were compared with those of the control groups for efficacy evaluation.

### 4.16 Determination of Organ Toxicity and Immunogenicity

On day seven after mRNA‐LNP administration, peripheral blood was collected from each group of mice, and plasma was obtained. ALT, AST, ALP, BUN, TNF‐α, and IL‐1β levels were measured using enzyme‐linked immunosorbent assay (ELISA) kits according to the manufacturer's instructions. The kits used were as follows: ALT (Biosen, BES1368K), AST (GIVEI, JW‐G21534), ALP (GIVEI, JW‐G20406), BUN (GIVEI, JW.MO1812), TNF‐α (GIVEI, JW‐G20852), and IL‐1β (GIVEI, JW‐G20174).

### 4.17 Histopathological Analysis

Tissue samples collected from various organs of mice in each group were fixed in 4% paraformaldehyde, routinely embedded in paraffin, and sectioned into 4 µm‐thick slices. The sections were stained following the standard protocol using a Hematoxylin and Eosin (H and E) Staining Kit (Yeasen, 60524ES60). The stained sections were then imaged under an optical microscope and subjected to histopathological analysis.

### 4.18 Statistical Analysis

All statistical analyses were performed using GraphPad Prism 9 software.

Data processing: Normalization was performed using internal references (such as Renilla luciferase activity, β‐actin mRNA/protein) to minimize variations from transfection efficiency, sample loading, and experimental batches.Quantitative results are expressed as mean ± standard deviation (S.D.).

Sample numbers (n): In vitro experiments were performed with n ≥ 3 independent biological replicates. In vivo experiments used n = 4 or n = 5 mice per group, as indicated in the corresponding figure legends.

Statistical tests: Comparisons between two groups were performed using two‐tailed unpaired Student's t‐test. Comparison among three or more groups was performed using one‐way analysis of variance (one‐way ANOVA) followed by Tukey's multiple comparisons test as post‐hoc analysis. The significance level was set at 0.05. Differences were considered statistically significant at *p* < 0.05.

## Funding

This work was supported by the Natural Science Foundation of China (22504136 to X.T.), the Natural Science Foundation of Anhui Province (2508085QH286 to X.T.), the Fundamental Research Funds for the Central Universities (WK9990250175 to X.T.), the New Cornerstone Science Foundation, and the Beijing Life Science Academy Initiative Scientific Research Program (2024101RPIA02 to J. L.).

## Conflicts of Interest

The authors declare no conflict of interest.

## Supporting information




**Supporting File**: advs75244‐sup‐0001‐SuppMat.docx.

## Data Availability

The data that support the findings of this study are available from the corresponding author upon reasonable request.
