# Supplementary material for: Enhanced Intracellular Stability and Translation Efficiency of mRNA Drugs by a 2‐arm mRNA Platform
Source: Adv Sci (Weinh). 2026 Apr 9:e75244. Online ahead of print. doi: 10.1002/advs.75244 (PMC13334632; doi:10.1002/advs.75244)
Supplement: Supplementary file 1 — Supporting File: advs75244‐sup‐0001‐SuppMat.docx. [file ADVS-9999-e75244-s001.docx]

Supporting Information

**Enhanced Intracellular Stability and Translation Efficiency of mRNA Drugs by a 2-arm mRNA Platform**

*Xucong Teng#,1,2, Jiahao Lin#,1, Qiushuang Zhang3, Xiangdong Zhang3, Yicong Dai*,1, Jinghong Li*,1,2,3*

1 Center for BioAnalytical Chemistry, Hefei National Laboratory of Physical Science at Microscale, University of Science and Technology of China, Hefei 230026, China

2 Beijing Life Science Academy, Beijing 102209, China.

3 New Cornerstone Science Laboratory, Department of Chemistry, Key Laboratory of Bioorganic Phosphorus Chemistry & Chemical Biology, Tsinghua University, Beijing 100084, China.

**S****upporting Information**

**Content**

*Supporting Information 1*

**Supplementary Figures 4**

*FIGURE S1 Qsep1 capillary electrophoresis characterization of 2-arm mRNA constructs. 4*

*FIGURE S2 Expression levels of dendritic mRNAs with different chemical and topological modifications. 5*

*FIGURE S3 Kinetic analysis of Firefly-degron (PEST) reporter mRNAs. 6*

*FIGURE S4 Electrophoretic mobility shift assay (EMSA) of the interaction between poly(A) tails and PABPC1. 7*

*FIGURE S5 Enhanced nuclease resistance of 2-arm poly(A). 8*

*FIGURE S6 The uncropped blots of Figure 4c. 9*

*FIGURE S7 Physicochemical characterization of mRNA-loaded lipid nanoparticles (LNPs). 10*

*FIGURE S8 2-arm hFVIII mRNA therapy enhances thrombosis to reduce spontaneous bleeding in FⅧ KO mice. 11*

*FIGURE S9 Plasma ALP and BUN levels of FⅧ KO mice after mRNA-LNP administration. 12*

*FIGURE S10 Liver and kidney injury biomarkers levels of wild-type mice after mRNA-LNP administration. 13*

*FIGURE S11 Histopathological analysis of tissue sections from major mouse organs after drug administration. 14*

**Supplementary Tables 15**

*Table S1. Information of mRNA sequences 15*

*Table S2. Information of oligos and chemical modifications 20*

Table S3. Information of shRNAs 22

**Supplementary Figures**

**
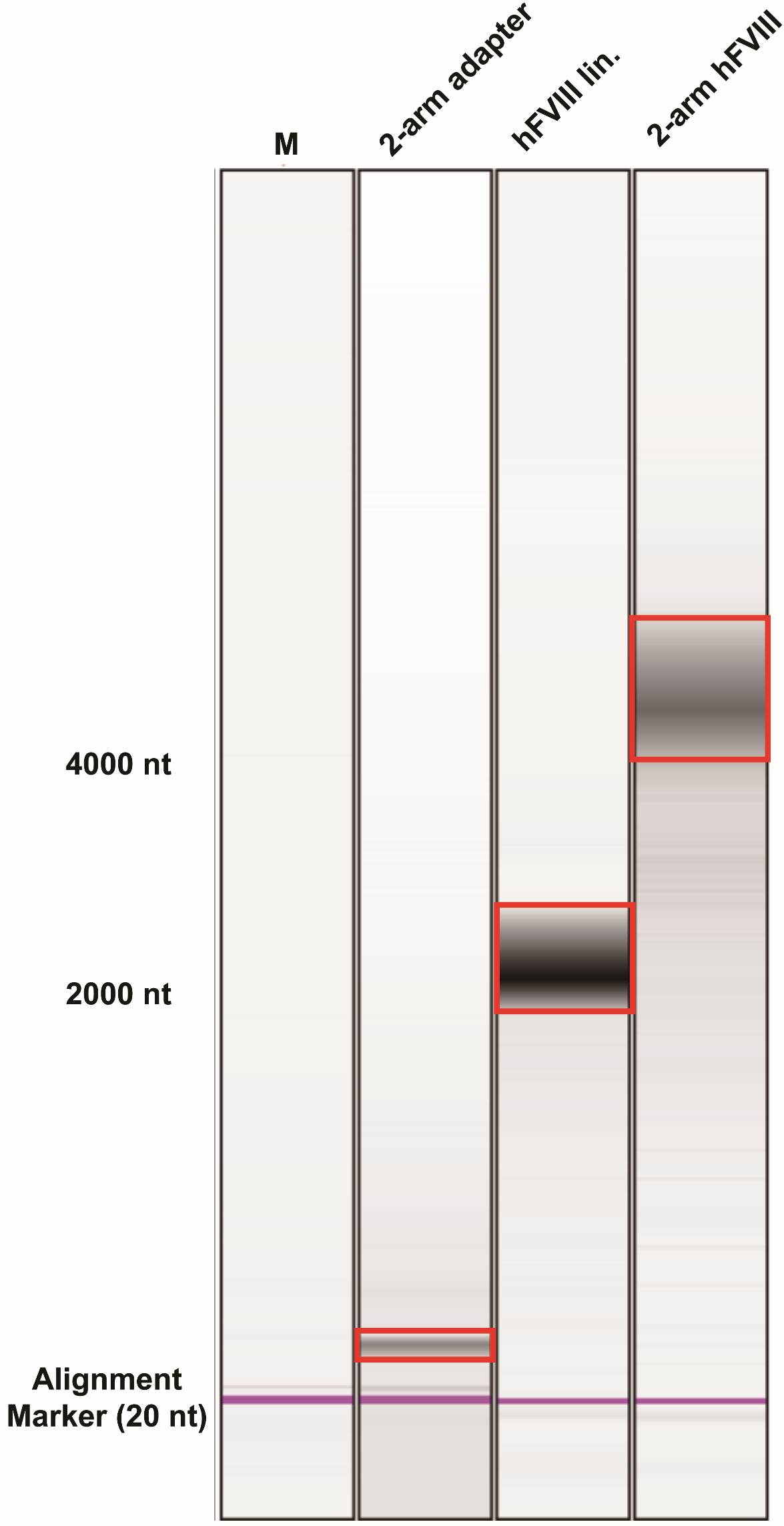
**

**FIGURE S1** **Qsep1 capillary electrophoresis characterization of 2-arm mRNA constructs.**

Representative capillary electrophoresis gel performed on the Qsep1 Bio-Fragment Analyzer to validate the purity and overall synthetic yield of the 2-arm mRNA constructs. Lane M: RNA alignment marker. The proportion of the target 2-arm FLuc mRNA was quantified using Image J. The expected band position of each product is highlighted with a red box. (n = 3, independent batches).

**
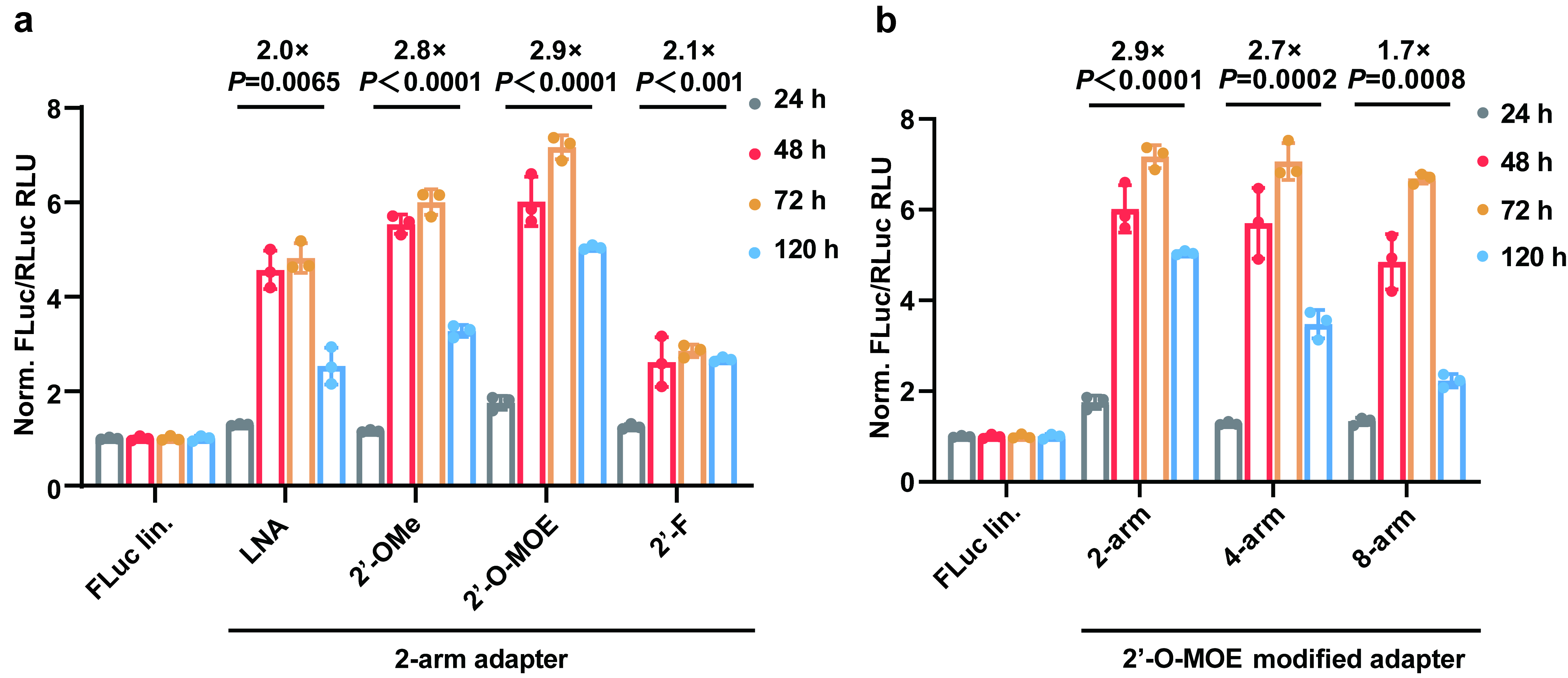
**

**FIGURE S2 Expression levels of dendritic mRNAs with different chemical and topological modifications.**

(a) Barplots of dendritic mRNAs with different unnatural nucleotides on the modified adapter. (b) Barplots of dendritic mRNAs consisted of different multi-arm adapters (2-arm, 4-arm and 8-arm). FLuc activity, normalized to RLuc and the linear FLuc mRNA control, were measured at 24, 48, 72 and 120 hours after transfection to quantify protein expression. Results were shown as mean ± s.d. (n = 3, independent replicates for each time point). The *P* values were evaluated by one-way ANOVA.


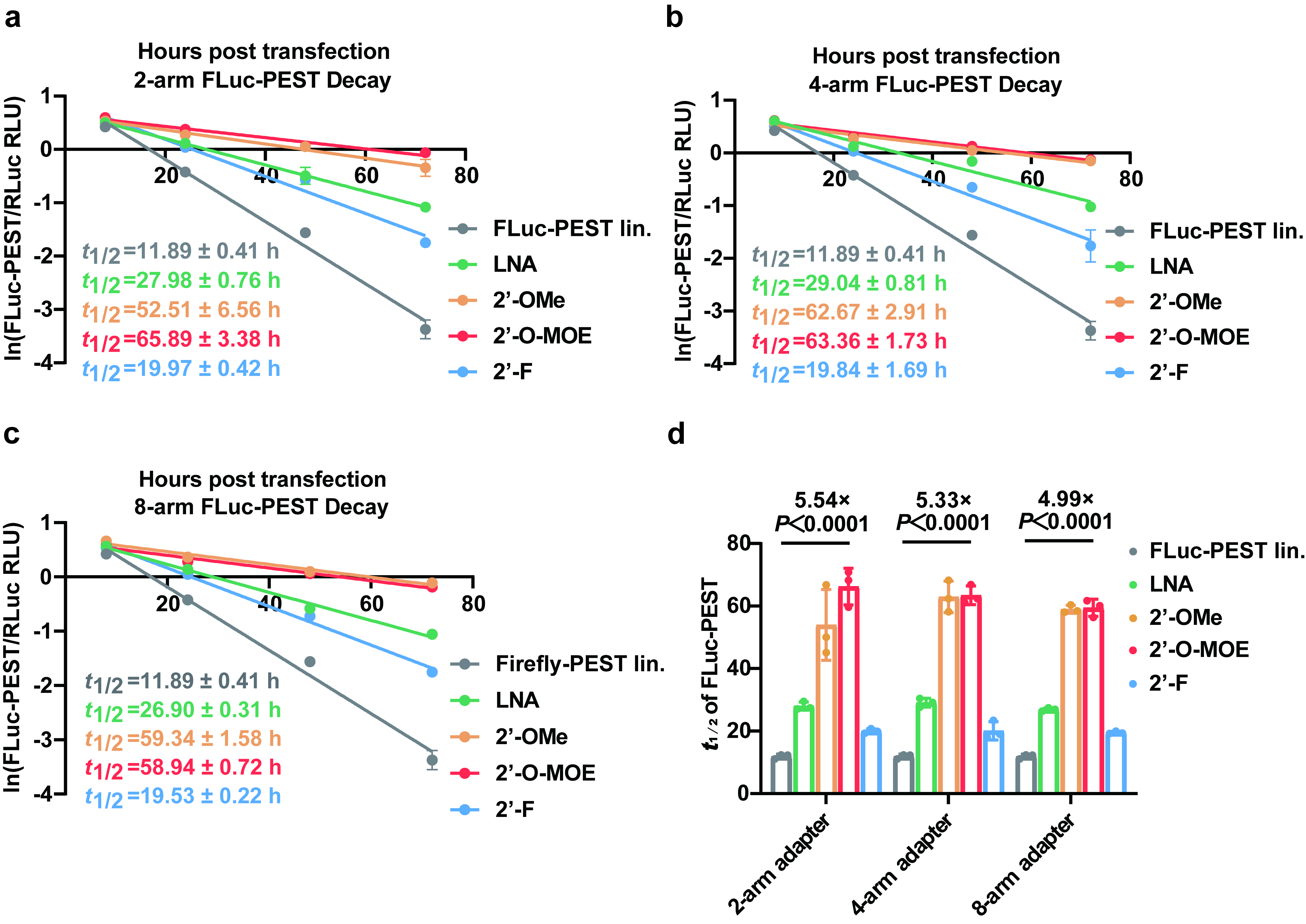


FIGURE S3 Kinetic analysis of Firefly-degron (PEST) reporter mRNAs.

(a) Degradation kinetics and half-life (*t*₁_/_₂) of dendritic mRNA with different 2-arm adapters. (b) Degradation kinetics and half-life (*t*₁_/_₂) of dendritic mRNA with different 4-arm adapters. (c) Degradation kinetics and half-life (*t*₁_/_₂) of dendritic mRNA with different 8-arm adapters. (d) Barplots of mRNA stability of different consturcts. Results were shown as mean ± s.d. (*n* = 3, independent replicates for each time point). The *P* values were evaluated by one-way ANOVA. The half-life (*t*₁/₂) of FLuc-PEST was determined by fitting the decay of normalized FLuc activity to a first-order kinetic model (lny = -kx + b), where k is the degradation rate obtained from the slope, and *t*₁_/_₂ was calculated as ln (2)/k.


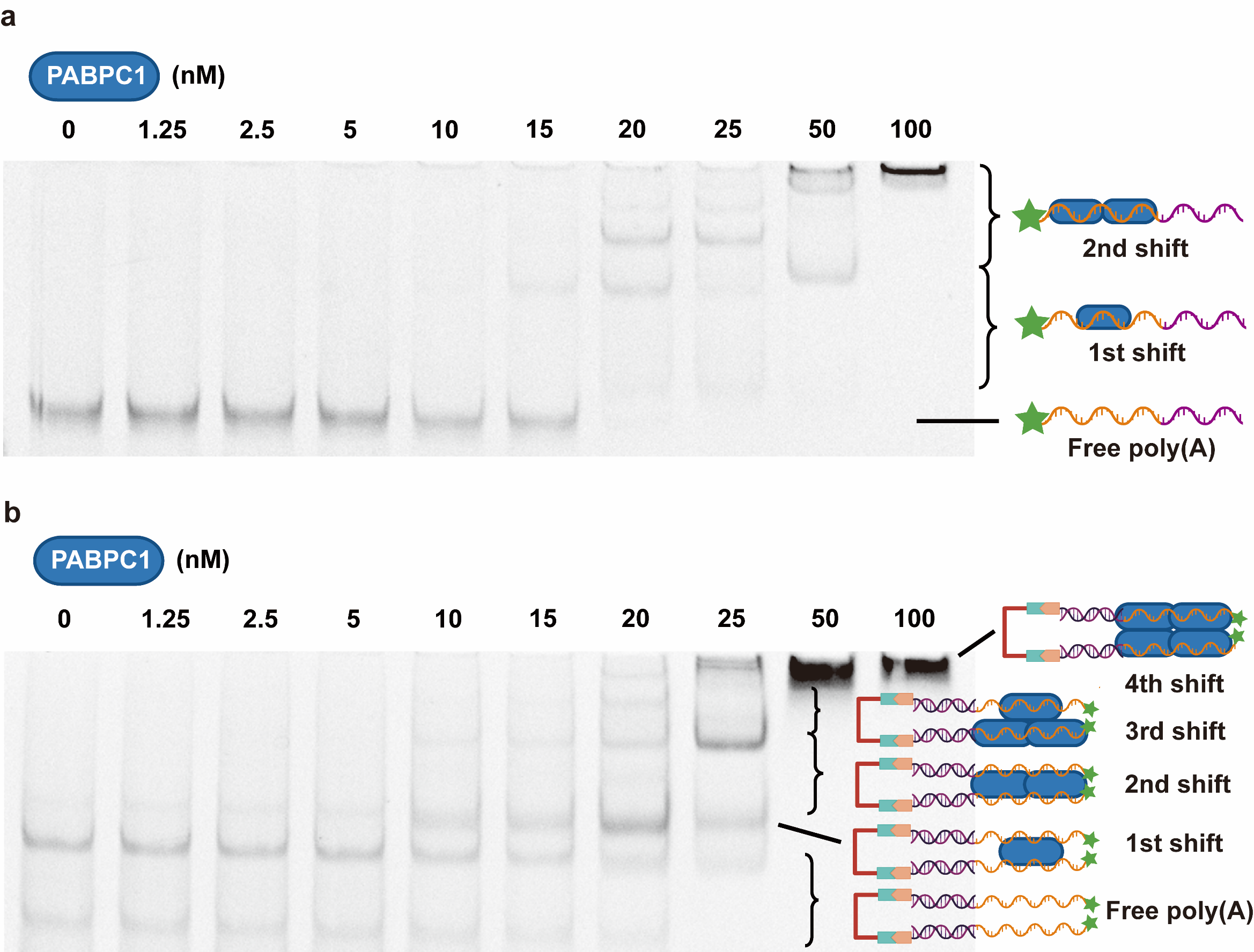


**FIGURE S****4** **Electrophoretic mobility shift assay (EMSA) of the interaction between** **poly(A) tails and PABPC1.**

(a) EMSA assay of linear AF488-rA_30_ binding with varying concentrations of recombinant human PABPC1 protein (PABPC1). The AF488-rA_30_ was 30 nt poly(A) labeled with Alexa Fluore 488 at 5’-end. (b) EMSA assay of 2-arm AF488-rA_30_ binding with varying concentrations of PABPC1, the same oligo with 2-arm adapter modification. 10% EMSA gel was used in this assay.


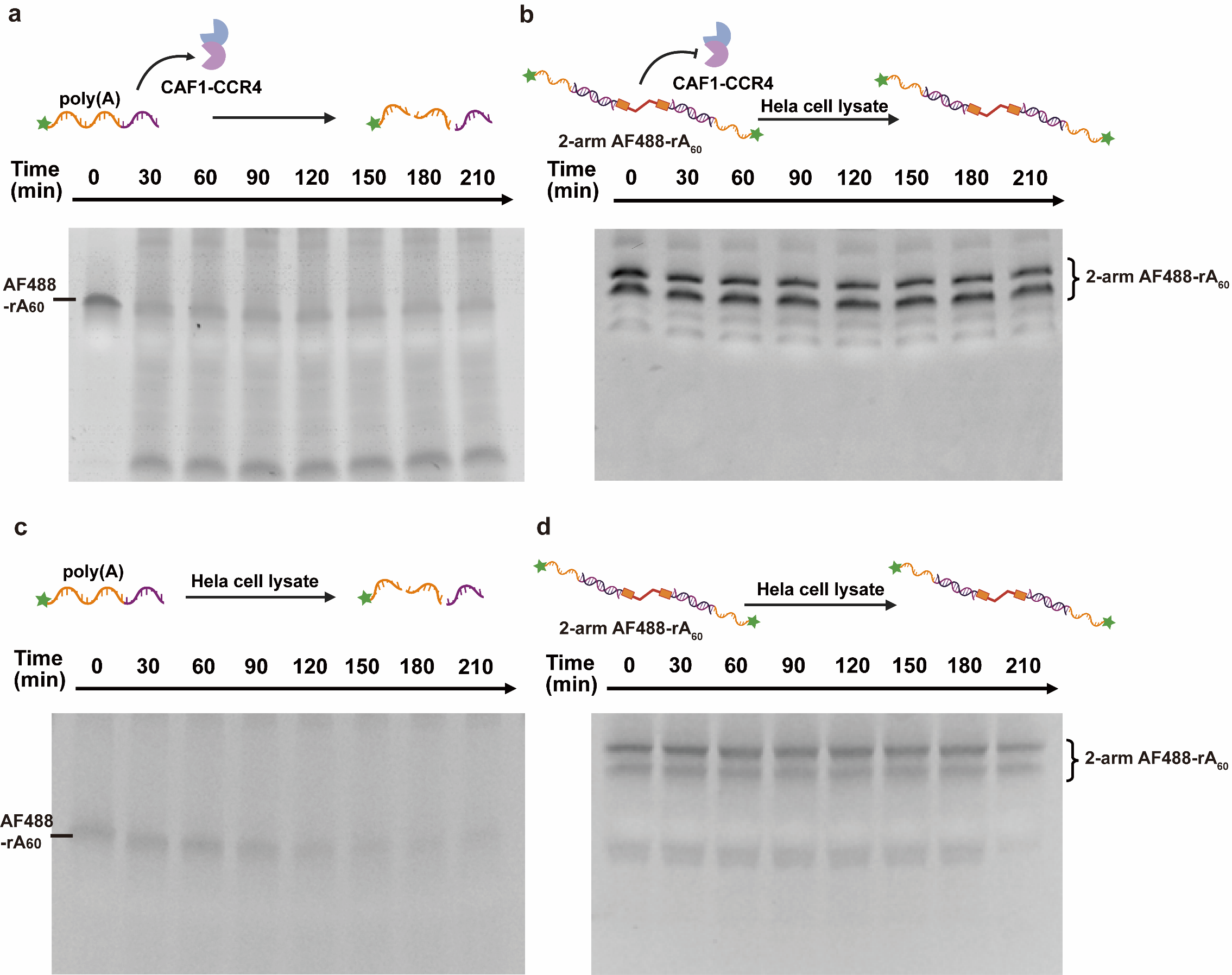


**FIGURE S5 Enhanced nuclease resistance of 2-arm poly(A).**

RNA stability and RNase deadenylation assay of two AF488-labeled poly(A) oligos: (a, c) linear AF488-rA_60_; (b, d) 2-arm AF488-rA_60_. Deadenylation process was monitored after treated with (a, b) recombinant CAF1/CCR4 complex for 210 min in vitro and (c, d) HeLa cytosolic lysate for 210 min in vitro. 15 % Urea-TBE PAGE was used in this assay.


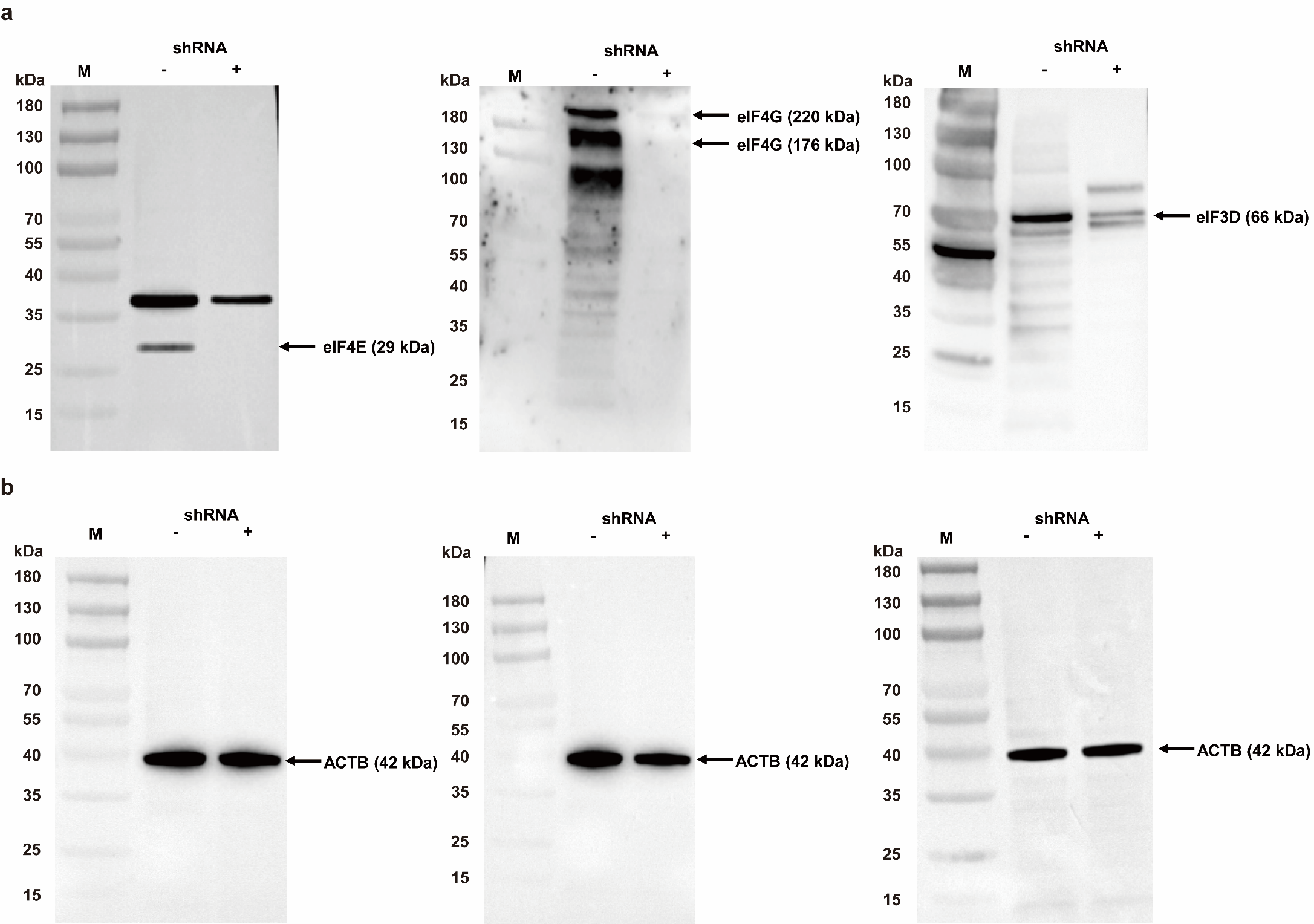


**FIGURE S6** **The** **uncropped blots of Figure 4c.**

(a) Uncropped blots of eIF4E/eIF4G/eIF3D in wild-type Hela cells (WT) and eIF4E/eIF4G/eIF3D KD Hela cells. (b) Uncropped blots of ACTB in wild-type Hela cells (WT) and eIF4E/eIF4G/eIF3D KD Hela cells.


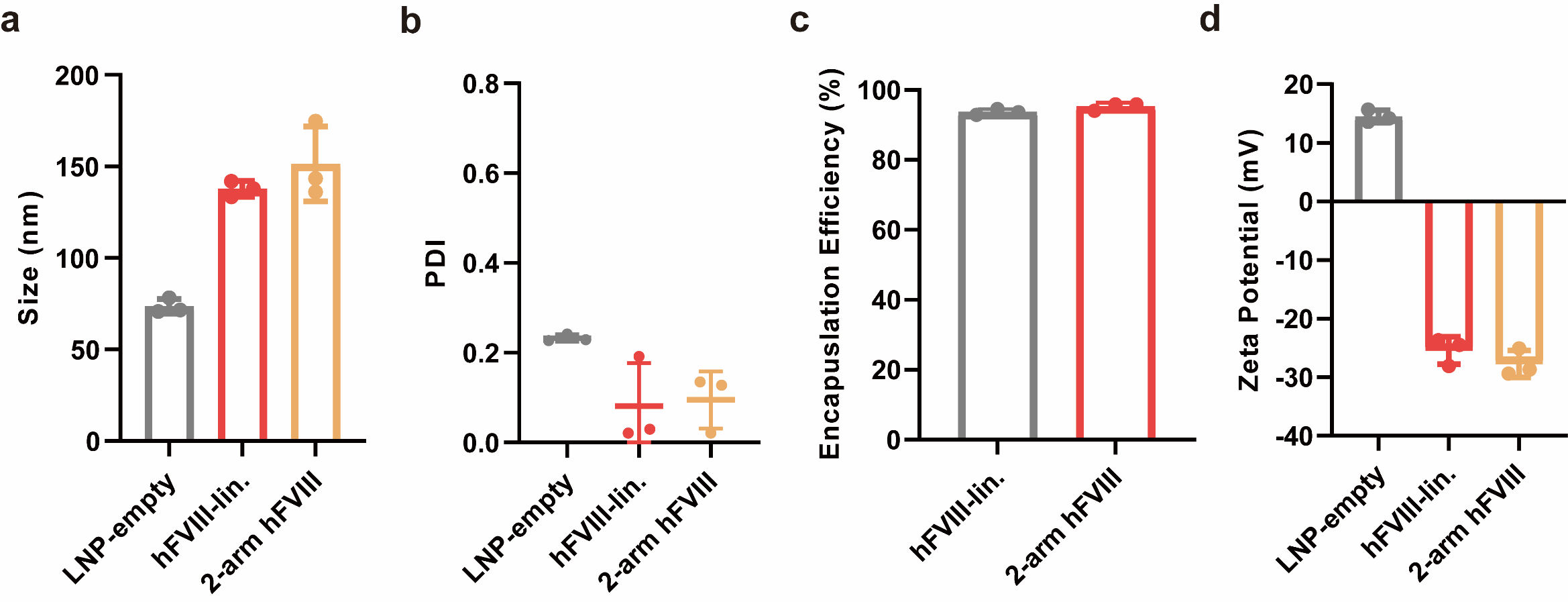


**FIGURE S7 Physicochemical characterization of mRNA-loaded lipid nanoparticles (LNPs).**

(a) Hydrodynamic diameter, (b) polydispersity index (PDI), (c) mRNA encapsulation efficiency, and (d) zeta potential of empty LNPs, linear hFVIII mRNA-loaded LNPs (hFVIII lin.), and 2-arm hFVIII mRNA-loaded LNPs (2-arm hFVIII). Particle size, PDI and zeta potential were measured by dynamic light scattering; mRNA encapsulation efficiency was quantified via RiboGreen fluorescence assay. Data are shown as mean ± s.d. (n = 3, independent batches).


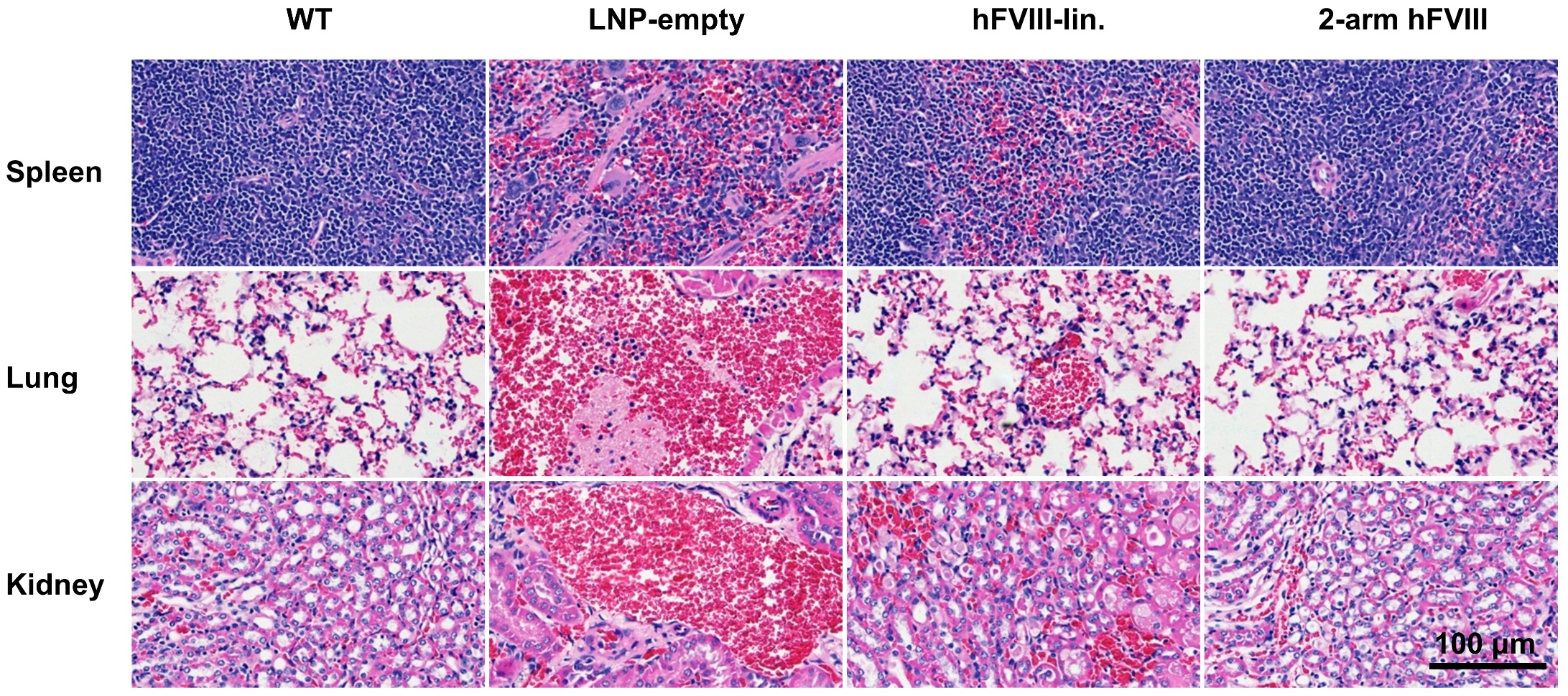


FIGURE S8 2-arm hFVIII mRNA therapy enhances thrombosis to reduce spontaneous bleeding in FⅧ KO mice.

Representative graphs of tissue sections from WT (Wild-type mice, untreated), LNP-empty (FⅧ KO mice, empty LNP treated), hFⅧ-lin. (FⅧ KO mice, linear F8 mRNA treated) and 2-arm hFⅧ (FⅧ KO mice, 2-arm F8 mRNA treated). Scale bar, 100 µm.


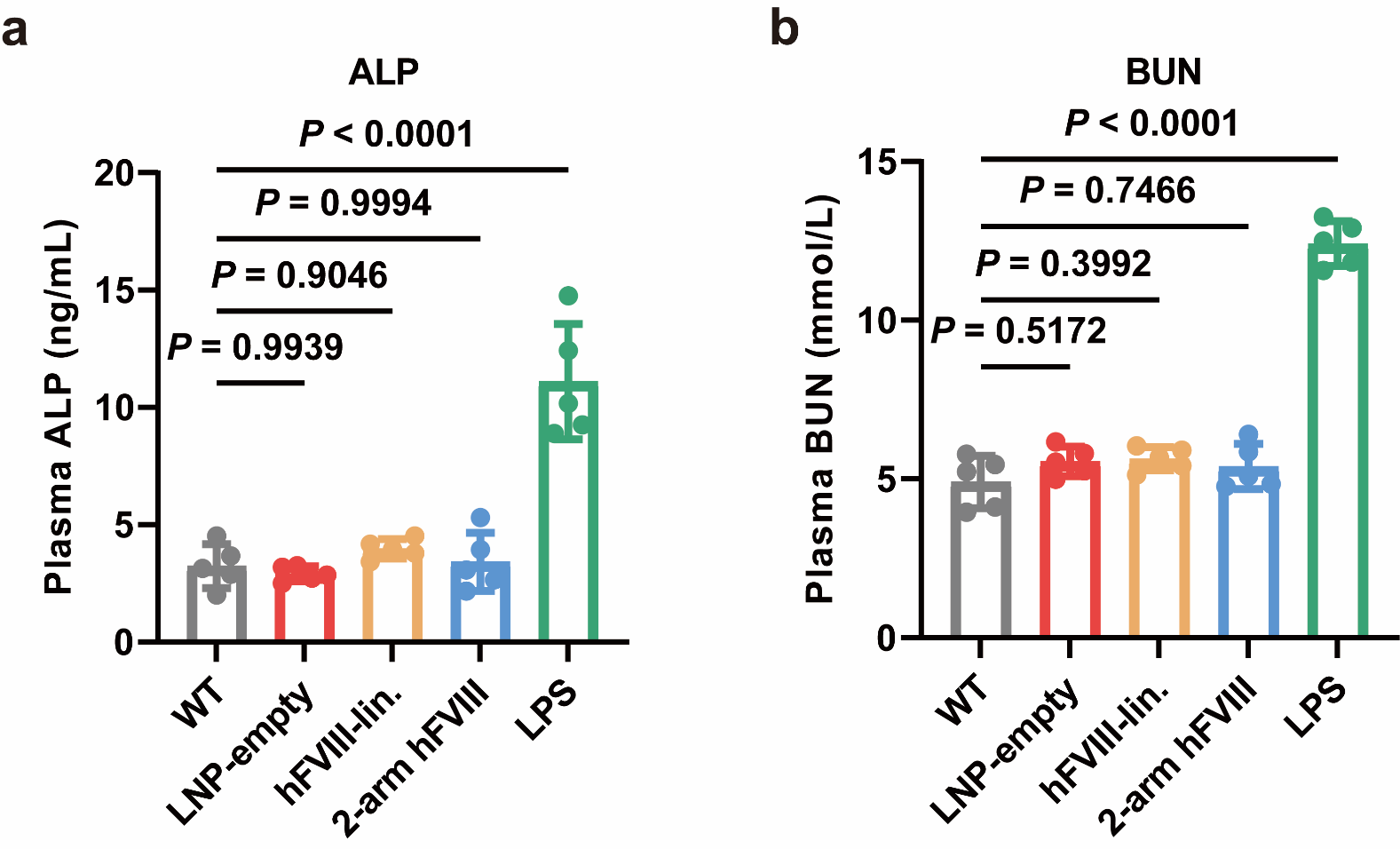


**FIGURE S9 Plasma ALP and BUN levels of** **F****Ⅷ KO mice after mRNA-LNP administration.**

(a) Plasma ALP concentration and, (b) plasma BUN concentration in FⅧ KO mice treated with empty LNPs, linear hFVIII mRNA (hFVIII lin.), 2-arm hFVIII mRNA (2-arm hFVIII), or LPS (positive control). Wild-type C57BL/6J mice were used as the negative control. The *P* values were calculated by one-way ANOVA. Data are presented as mean ± s.d. (n=5 mice per group).


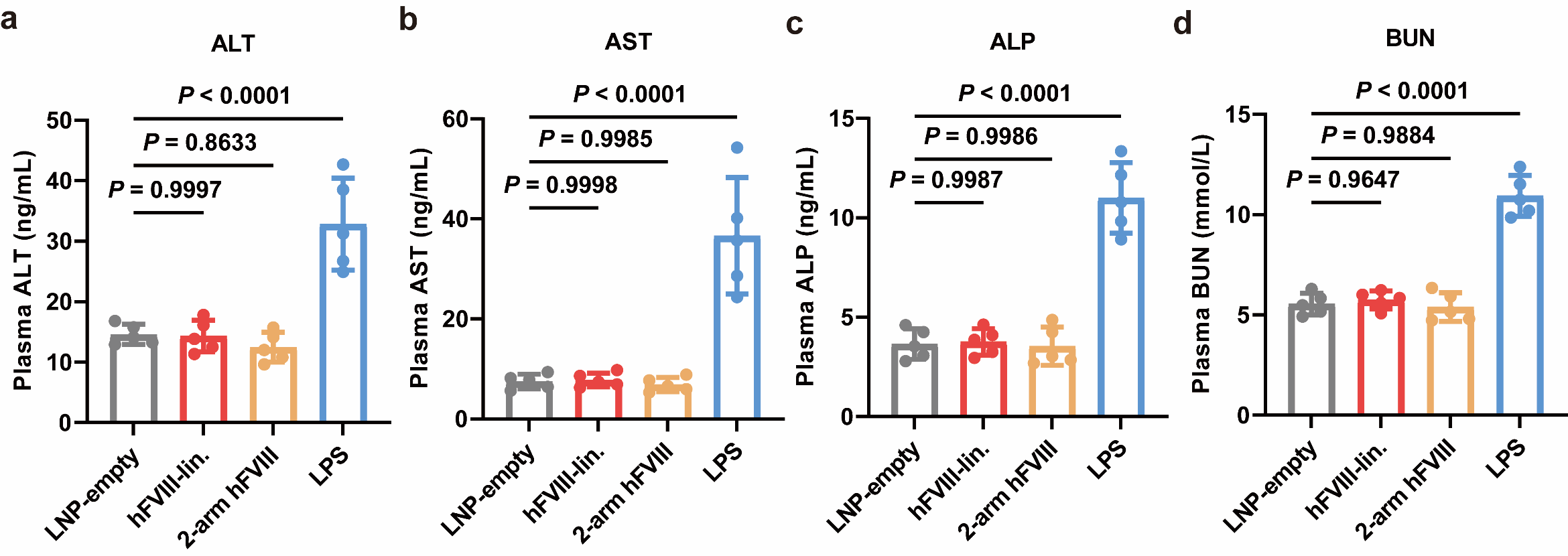


**FIGURE** **S10 Liver and kidney injury biomarkers levels of wild-type mice after mRNA-LNP administration.**

Plasma levels of (a) ALT, (b) AST, (c) ALP, and (d) BUN in wild-type C57BL/6J mice treated with empty LNP, linear hFVIII mRNA (hFVIII lin.), 2-arm hFVIII mRNA (2-arm hFVIII), or LPS (for organ injury). The *P* values were calculated by one-way ANOVA. Data are presented as mean ± s.d. (n=5 mice per group).


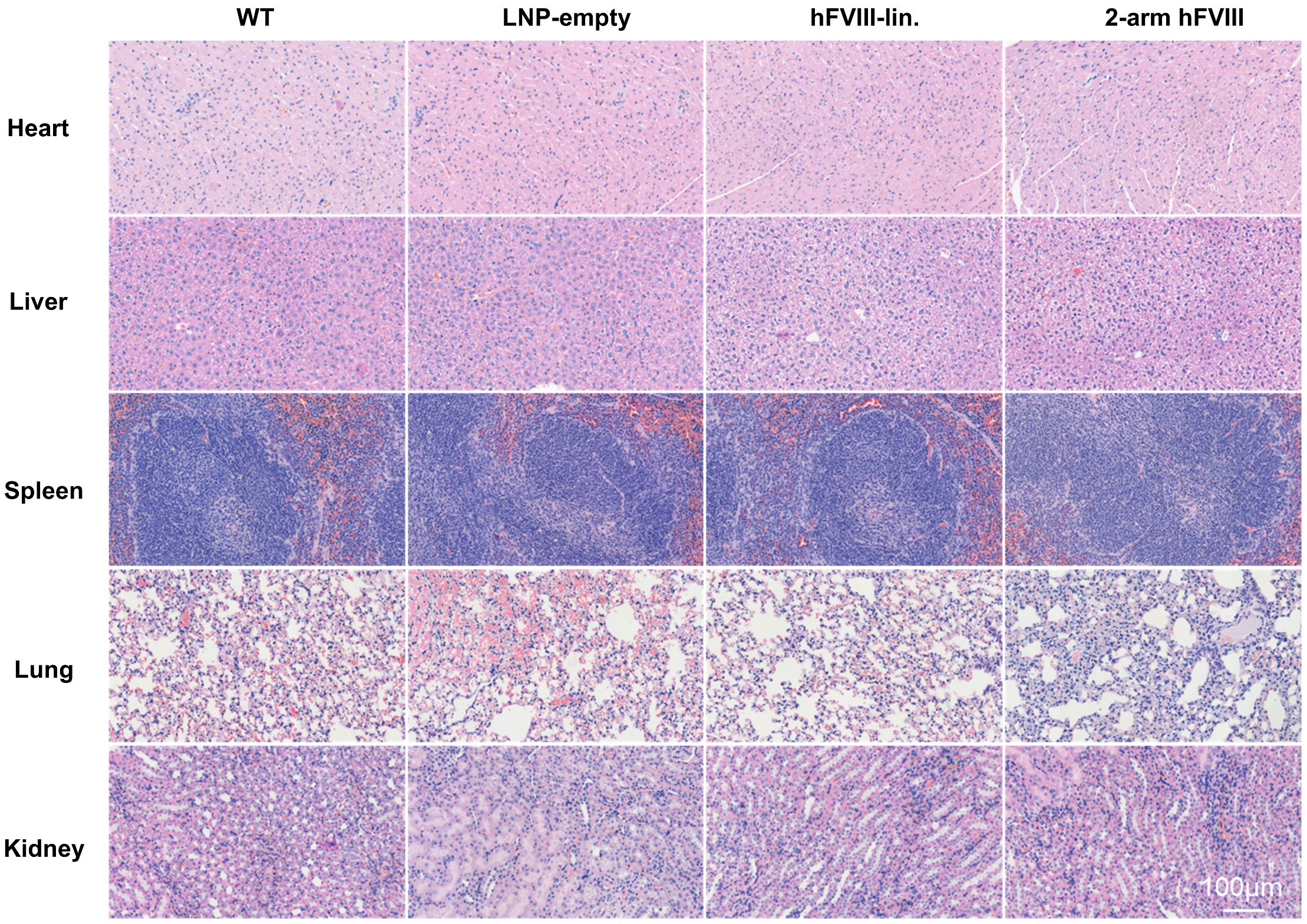


**FIGURE** **S11** **Histopathological analysis of tissue sections from major mouse organs after drug administration.**

Scale bar, 100 µm.

**Supplementary Tables**

**Table S1.** **Information of mRNA** **sequences**

| **Name** | **Sequences (5’ to 3’)** | **Note** |
| --- | --- | --- |
| Firefly Luciferase | GAGAAUAAACUAGUAUUCUUCUGGUCCCCACAGACUCAGAGAGAACCCGCCACCAUGGAAGAUGCCAAAAACAUUAAGAAGGGCCCAGCGCCAUUCUACCCACUCGAAGACGGGACCGCCGGCGAGCAGCUGCACAAAGCCAUGAAGCGCUACGCCCUGGUGCCCGGCACCAUCGCCUUUACCGACGCACAUAUCGAGGUGGACAUUACCUACGCCGAGUACUUCGAGAUGAGCGUUCGGCUGGCAGAAGCUAUGAAGCGCUAUGGGCUGAAUACAAACCAUCGGAUCGUGGUGUGCAGCGAGAAUAGCUUGCAGUUCUUCAUGCCCGUGUUGGGUGCCCUGUUCAUCGGUGUGGCUGUGGCCCCAGCUAACGACAUCUACAACGAGCGCGAGCUGCUGAACAGCAUGGGCAUCAGCCAGCCCACCGUCGUAUUCGUGAGCAAGAAAGGGCUGCAAAAGAUCCUCAACGUGCAAAAGAAGCUACCGAUCAUACAAAAGAUCAUCAUCAUGGAUAGCAAGACCGACUACCAGGGCUUCCAAAGCAUGUACACCUUCGUGACUUCCCAUUUGCCACCCGGCUUCAACGAGUACGACUUCGUGCCCGAGAGCUUCGACCGGGACAAAACCAUCGCCCUGAUCAUGAACAGUAGUGGCAGUACCGGAUUGCCCAAGGGCGUAGCCCUACCGCACCGCACCGCUUGUGUCCGAUUCAGUCAUGCCCGCGACCCCAUCUUCGGCAACCAGAUCAUCCCCGACACCGCUAUCCUCAGCGUGGUGCCAUUUCACCACGGCUUCGGCAUGUUCACCACGCUGGGCUACUUGAUCUGCGGCUUUCGGGUCGUGCUCAUGUACCGCUUCGAGGAGGAGCUAUUCUUGCGCAGCUUGCAAGACUAUAAGAUUCAAUCUGCCCUGCUGGUGCCCACACUAUUUAGCUUCUUCGCUAAGAGCACUCUCAUCGACAAGUACGACCUAAGCAACUUGCACGAGAUCGCCAGCGGCGGGGCGCCGCUCAGCAAGGAGGUAGGUGAGGCCGUGGCCAAACGCUUCCACCUACCAGGCAUCCGCCAGGGCUACGGCCUGACAGAAACAACCAGCGCCAUUCUGAUCACCCCCGAAGGGGACGACAAGCCUGGCGCAGUAGGCAAGGUGGUGCCCUUCUUCGAGGCUAAGGUGGUGGACUUGGACACCGGUAAGACACUGGGUGUGAACCAGCGCGGCGAGCUGUGCGUCCGUGGCCCCAUGAUCAUGAGCGGCUACGUUAACAACCCCGAGGCUACAAACGCUCUCAUCGACAAGGACGGCUGGCUGCACAGCGGCGACAUCGCCUACUGGGACGAGGACGAGCACUUCUUCAUCGUGGACCGGCUGAAGAGCCUGAUCAAAUACAAGGGCUACCAGGUAGCCCCAGCCGAACUGGAGAGCAUCCUGCUGCAACACCCCAACAUCUUCGACGCCGGGGUCGCCGGCCUGCCCGACGACGAUGCCGGCGAGCUGCCCGCCGCAGUCGUCGUGCUGGAACACGGUAAAACCAUGACCGAGAAGGAGAUCGUGGACUAUGUGGCCAGCCAGGUUACAACCGCCAAGAAGCUGCGCGGUGGUGUUGUGUUCGUGGACGAGGUGCCUAAAGGACUGACCGGCAAGUUGGACGCCCGCAAGAUCCGCGAGAUUCUCAUUAAGGCCAAGAAGGGCGGCAAGAUCGCCGUGGCUGGAGCCUCGGUGGCCAUGCUUCUUGCCCCUUGGGCCUCCCCCCAGCCCCUCCUCCCCUUCCUGCACCCGUACCCCCGUGGUCUUUGAAUAAAGUCUGAGUGGGCGGCAAAAAAAAAAAAAAAAAAAAAAAAAAAAAAAGCAUAUGACUAAAAAAAAAAAAAAAAAAAAAAAAAAAAAAAAAAAAAAAAAAAAAAAAAAAAAAAAAAAAAAAAAAAAAACUUAAGCUUGGUACCGAGCUCGGAUC | Yellow:5’-UTR    Red: CDS of Firefly Luciferase      Green: 3’-UTR  Pink: Poly(A) tail  Teal: Adapter binding sequence |
| Firefly Luciferase-PEST | GAGAAUAAACUAGUAUUCUUCUGGUCCCCACAGACUCAGAGAGAACCCGCCACCAUGGAAGAUGCCAAAAACAUUAAGAAGGGCCCAGCGCCAUUCUACCCACUCGAAGACGGGACCGCCGGCGAGCAGCUGCACAAAGCCAUGAAGCGCUACGCCCUGGUGCCCGGCACCAUCGCCUUUACCGACGCACAUAUCGAGGUGGACAUUACCUACGCCGAGUACUUCGAGAUGAGCGUUCGGCUGGCAGAAGCUAUGAAGCGCUAUGGGCUGAAUACAAACCAUCGGAUCGUGGUGUGCAGCGAGAAUAGCUUGCAGUUCUUCAUGCCCGUGUUGGGUGCCCUGUUCAUCGGUGUGGCUGUGGCCCCAGCUAACGACAUCUACAACGAGCGCGAGCUGCUGAACAGCAUGGGCAUCAGCCAGCCCACCGUCGUAUUCGUGAGCAAGAAAGGGCUGCAAAAGAUCCUCAACGUGCAAAAGAAGCUACCGAUCAUACAAAAGAUCAUCAUCAUGGAUAGCAAGACCGACUACCAGGGCUUCCAAAGCAUGUACACCUUCGUGACUUCCCAUUUGCCACCCGGCUUCAACGAGUACGACUUCGUGCCCGAGAGCUUCGACCGGGACAAAACCAUCGCCCUGAUCAUGAACAGUAGUGGCAGUACCGGAUUGCCCAAGGGCGUAGCCCUACCGCACCGCACCGCUUGUGUCCGAUUCAGUCAUGCCCGCGACCCCAUCUUCGGCAACCAGAUCAUCCCCGACACCGCUAUCCUCAGCGUGGUGCCAUUUCACCACGGCUUCGGCAUGUUCACCACGCUGGGCUACUUGAUCUGCGGCUUUCGGGUCGUGCUCAUGUACCGCUUCGAGGAGGAGCUAUUCUUGCGCAGCUUGCAAGACUAUAAGAUUCAAUCUGCCCUGCUGGUGCCCACACUAUUUAGCUUCUUCGCUAAGAGCACUCUCAUCGACAAGUACGACCUAAGCAACUUGCACGAGAUCGCCAGCGGCGGGGCGCCGCUCAGCAAGGAGGUAGGUGAGGCCGUGGCCAAACGCUUCCACCUACCAGGCAUCCGCCAGGGCUACGGCCUGACAGAAACAACCAGCGCCAUUCUGAUCACCCCCGAAGGGGACGACAAGCCUGGCGCAGUAGGCAAGGUGGUGCCCUUCUUCGAGGCUAAGGUGGUGGACUUGGACACCGGUAAGACACUGGGUGUGAACCAGCGCGGCGAGCUGUGCGUCCGUGGCCCCAUGAUCAUGAGCGGCUACGUUAACAACCCCGAGGCUACAAACGCUCUCAUCGACAAGGACGGCUGGCUGCACAGCGGCGACAUCGCCUACUGGGACGAGGACGAGCACUUCUUCAUCGUGGACCGGCUGAAGAGCCUGAUCAAAUACAAGGGCUACCAGGUAGCCCCAGCCGAACUGGAGAGCAUCCUGCUGCAACACCCCAACAUCUUCGACGCCGGGGUCGCCGGCCUGCCCGACGACGAUGCCGGCGAGCUGCCCGCCGCAGUCGUCGUGCUGGAACACGGUAAAACCAUGACCGAGAAGGAGAUCGUGGACUAUGUGGCCAGCCAGGUUACAACCGCCAAGAAGCUGCGCGGUGGUGUUGUGUUCGUGGACGAGGUGCCUAAAGGACUGACCGGCAAGUUGGACGCCCGCAAGAUCCGCGAGAUUCUCAUUAAGGCCAAGAAGGGCGGCAAGAUCGCCGUGUCUCACGGCUTUCCGCCTGAGGUUGAAGAGCAAGCCGCCGGUACAUUGCCUAUGUCCUGCGCACAAGAAAGCGGUAUGGACCGGCACCCAGCCGCUUGUGCUUCAGCUCGCAUCAACGUCGCUGGAGCCUCGGUGGCCAUGCUUCUUGCCCCUUGGGCCUCCCCCCAGCCCCUCCUCCCCUUCCUGCACCCGUACCCCCGUGGUCUUUGAAUAAAGUCUGAGUGGGCGGCAAAAAAAAAAAAAAAAAAAAAAAAAAAAAAAGCAUAUGACUAAAAAAAAAAAAAAAAAAAAAAAAAAAAAAAAAAAAAAAAAAAAAAAAAAAAAAAAAAAAAAAAAAAAAACUUAAGCUUGGUACCGAGCUCGGAUC | Yellow: 5’-UTR  Red: CDS of Firefly Luciferase-PEST  Green: 3’-UTR  Pink: Poly(A) tail  Teal: Adapter binding sequence |
| Renilla Luciferase | GAGACCCAAGCUGGCUAGCGCCACCAUGACCAGCAAAGUGUAUGAUCCGGAACAGCGCAAACGCAUGAUUACCGGCCCGCAGUGGUGGGCGCGCUGCAAACAGAUGAACGUGCUGGAUAGCUUUAUUAACUAUUAUGAUAGCGAAAAACAUGCGGAAAACGCGGUGAUUUUUCUGCAUGGCAACGCGGCGAGCAGCUAUCUGUGGCGGCAUGUGGUGCCGCAUAUUGAACCAGUGGCGCGCUGCAUUAUUCCGGAUCUGAUUGGCAUGGGCAAAAGCGGCAAAAGCGGCAACGGCAGCUAUCGCCUGCUGGAUCAUUAUAAAUAUCUGACCGCGUGGUUUGAACUGCUGAACCUGCCGAAAAAAAUUAUUUUUGUGGGCCAUGAUUGGGGCGCGUGCCUGGCGUUUCAUUAUAGCUAUGAACAUCAGGAUAAAAUUAAAGCGAUUGUGCACGCGGAAAGCGUGGUGGAUGUGAUUGAAAGCUGGGAUGAAUGGCCGGAUAUUGAAGAAGAUAUUGCGCUGAUUAAAAGCGAAGAAGGCGAAAAAAUGGUGCUGGAAAACAACUUUUUUGUGGAAACCAUGCUGCCGAGCAAAAUUAUGCGCAAACUGGAACCGGAAGAAUUUGCGGCGUAUCUGGAACCGUUUAAAGAAAAAGGCGAAGUGCGCCGCCCGACCCUGAGCUGGCCGCGCGAAAUUCCGCUGGUGAAAGGCGGCAAACCGGAUGUGGUCCAGAUUGUGCGCAACUAUAACGCGUAUCUGCGCGCGAGCGAUGAUCUGCCGAAAAUGUUUAUUGAAAGCGAUCCGGGCUUUUUUAGCAACGCGAUUGUGGAAGGCGCGAAAAAAUUUCCGAACACCGAAUUUGUGAAAGUGAAGGGCCUGCAUUUUAGCCAGGAAGAUGCGCCGGAUGAAAUGGGCAAAUAUAUUAAGAGCUUUGUGGAACGCGUGCUGAAAAACGAACAGGGCUAUCUCACGGCUUUCCGCCUGAGGUUGAAGAGCAAGCCGCCGGUACAUUGCCUAUGUCCUGCGCACAAGAAAGCGGUAUGGACCGGCACCCAGCCGCUUGUGCUUCAGCUCGCAUCAACGUCUAAAAAAAAAAAAAAAAAAAAAAAAAAAAAAAAGCAUAUGACUAAAAAAAAAAAAAAAAAAAAAAAAAAAAAAAAAAAAAAAAAAAAAAAAAAAAAAAAAAAAAAAAAAAAAA | Yellow: 5’-UTR  Red: CDS of Renilla Luciferase    Pink: Poly(A) tail |
| Homo sapiens coagulation factor VIII (F8), transcript variant 1 | GAGAAUAAACUAGUAUUCUUCUGGUCCCCACAGACUCAGAGAGAACCCGCCACC  -CDS-GCUGGAGCCUCGGUGGCCAUGCUUCUUGCCCCUUGGGCCUCCCCCCAGCCCCUCCUCCCCUUCCUGCACCCGUACCCCCGUGGUCUUUGAAUAAAGUCUGAGUGGGCGGCAAAAAAAAAAAAAAAAAAAAAAAAAAAAAAAGCAUAUGACUAAAAAAAAAAAAAAAAAAAAAAAAAAAAAAAAAAAAAAAAAAAAAAAAAAAAAAAAAAAAAAAAAAAAAACUUAAGCUUGGUACCGAGCUCGGAUC | Yellow: 5’-UTR  Red: CDS see NM_000132.4 in NCBI  Green：3’-UTR  Pink: Poly(A) tail  Teal: Adapter binding sequence |

**Table S2. Information of oligos and chemical modifications**

| **Name** | **Sequences (5’ to 3’)** | **Note** |
| --- | --- | --- |
| AF488-rA_60_ | /5Alex488N/AAAAAAAAAAAAAAAAAAAAAAAAAAAAAAAAAAAAAAAAAAAAAAAAAAAAAAAAAAAACUUAAGCUUGGUACCGAGCUCGGAUC | Oligo is modified with Alexa Fluor 488 at 5’-end; all nucleotides are ribonucleotides |
| AF488-rA_30_ | /5Alex488N/AAAAAAAAAAAAAAAAAAAAAAAAAAAAAACUUAAGCUUGGUACCGAGCUCGGAUC | Oligo is modified with Alexa Fluor 488 at 5’-end; all nucleotides are ribonucleotides |
| 5’Azide-modified adapter | /5AzideN/GATCCGAGCTCGGTACCAAG | Oligo is modified with azide at 5’-end; all nucleotides are deoxyribonucleotides |
| 5’Azide-modified LNA adapter | /5AzideN/GAUCCGAGCUCGGUACCAAG | Oligo is modified with azide at 5’-end; all nucleotides are locked ribonucleotides |
| 5’Azide-modified 2’-O-MOE adapter | /5AzideN/GAUCCGAGCUCGGUACCAAG | Oligo is modified with azide at 5’-end; all nucleotides are 2’-O-MOE modified ribonucleotides |
| 5’Azide-modified 2’-OMe adapter | /5AzideN/GAUCCGAGCUCGGUACCAAG | Oligo is modified with azide at 5’-end; all nucleotides are 2’-OMe modified ribonucleotides |
| 5’Azide-modified 2’-F adapter | /5AzideN/GAUCCGAGCUCGGUACCAAG | Oligo is modified with azide at 5’-end; all nucleotides are 2’-F modified ribonucleotides |

**Table S3. Information of shRNAs**

| **Identifier** | **Sequences (5’ to 3’)** |
| --- | --- |
| eIF4E_shRNA | CCGGCCAAAGAUAGUGAUUGGUUAUCUCGAGAUAACCAAUCACUAUCUUUGGUUUUUG |
| eIF4G_shRNA | CCGGGCCCUUGUAGUGACCUUAGAACUCGAGUUCUAAGGUCACUACAAGGGCUUUUUG |
| eIF3D_shRNA | CCGGGACGACAUGGAUAAGAAUGAACUCGAGUUCAUUCUUAUCCAUGUCGUCUUUUUUG |
